# Anomalous Last Interglacial Tyrrhenian sea levels and Neanderthal settling at Guattari and Moscerini caves (central Italy)

**DOI:** 10.1038/s41598-020-68604-z

**Published:** 2020-07-17

**Authors:** F. Marra, M. F. Rolfo, M. Gaeta, F. Florindo

**Affiliations:** 1 Istituto Nazionale di Geofisica e Vulcanologia, Via di Vigna Murata 605, 00143 Rome, Italy; 2 Department of History, Humanities and Society, University of Rome “Tor Vergata”, Via Columbia 1, 00133 Rome, Italy; 3 Dipartimento di Scienze della Terra, “Sapienza” Università di Roma, Piazzale Aldo Moro 5, 00185 Rome, Italy; 4 Institute for Climate Change Solutions, via Sorchio, 61040 Frontone, Pesaro e Urbino Italy

**Keywords:** Climate sciences, Ocean sciences, Solid Earth sciences

## Abstract

We present a geological-stratigraphical study aimed to provide chronologic constraints to the sea-level markers occurring at two coastal caves of central Italy (Grotta Guattari and Grotta dei Moscerini) and to the Neanderthal frequentation of these caves, in the light of recent archaeological and geomorphological-geochronological studies suggesting similar sea levels during MIS 5.5 and MIS 5.3, and only few m below the Present during MIS 5.1 in this region. Based on the review of previous literature data, combined with new stratigraphic observations at Grotta Guattari and re-analysis of archive material including unpublished field notes from Grotta dei Moscerini, we reconstruct a plausible sea-level history accounting for the lithological and paleoenvironmental features of their sedimentary fillings. In particular, we outline the abundant occurrence of well-rounded pumice clasts within the sedimentary deposits of Moscerini Cave, attesting for the proximity to the beach where this pumice was gathered by wave action. Through the petrographic and geochemical analysis of this pumice we evidence provenance from Phlegraean Fields and Ischia Island volcanic districts, framing their chronology in the time span 118–40 ka, consistent with literature ESR-U/Th dates providing ages ranging 101 ± 5–74 ± 7 ka for the sedimentary filling of both Moscerini and Guattari caves.

## Introduction

**Figure 1:**
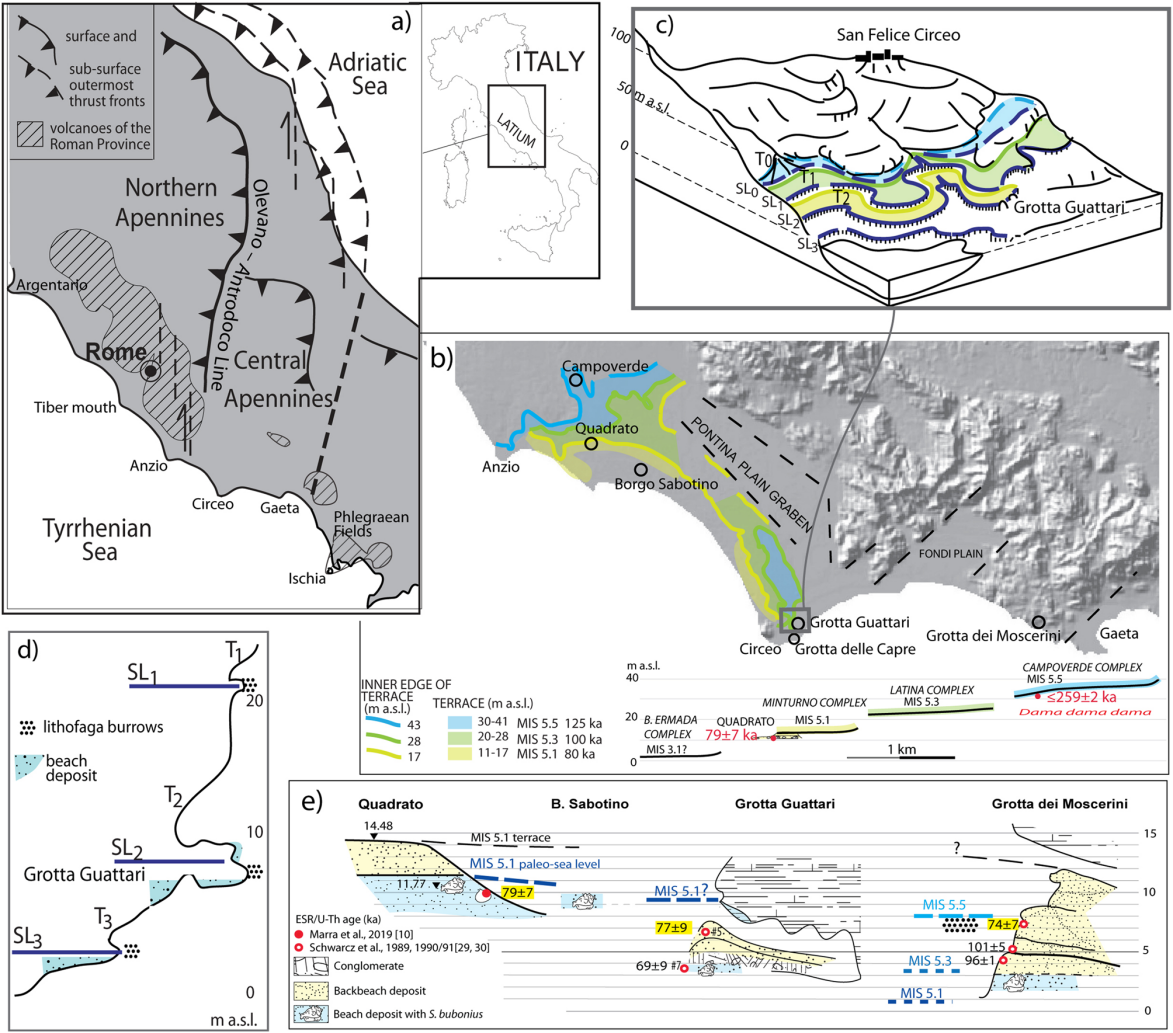
(**a**) Structural sketch of central Italy. (**b**) Digital Elevation Map (DEM) image of the southern Latium coast showing the suite of three marine terraces described in^10^. Background DEM: TINITALY/01 square WA 6,570, used with permission by the Istituto Nazionale di Geofisica e Vulcanologia, Rome. The cross-section shows the correlation among the marine terraces reconstructed by^19^ and those identified by^10^; bio- and geochronologic constraints (in red) allowing for correlation with MIS 5.5, MIS 5.3 and MIS 5.1 are also reported. (**c**) Coastlines (SL) and related terraces (T) reconstructed at Circeo promontory in the surrounding of Grotta Guattari by^21–23^. (**d**) sea-level indicators represented by tidal notches, lithophaga burrows and biodetritic beach deposits. (**e**) Correlation among different sea-level markers ranging 10–8 m a.s.l., represented by the terraced deposit of the Minturno complex in Quadrato, and by the beach to backbeach sedimentary filling of Grotta Guattari and Grotta dei Moscerini; the three consistent ESR/U-Th ages suggesting correlation with MIS 5.1 are shown. All drawings by F. Marra.

A series of caves which yielded an important record of Neanderthal remains, including an almost intact cranium^1^, along with a number of artifacts featuring the outstanding occurrence of instruments made on shell^2–4^, occur at elevation ranging 5–10 m a.s.l. along the carbonatic reefs forming the coast of the Tyrrhenian Sea in southern Latium (Fig. 1b). Frequentation of these caves has been considered to be possible in a systematic manner after the sea-level drop following the Last Interglacial stage (MIS 5.5). In contrast, during the highstand, circa 125 ka, it was assumed that the seawater entered the caves, producing a series of sea-level markers, represented by tidal notches, horizontal stripes of lithodomus burrows, and characteristic biodetritic beach deposits ("panchina"). Based on inferences provided by these sea-level markers occurring a few meters above the present sea level, this region has been considered to be substantially stable from a tectonic point of view. This is consistent with the common assumption of a maximum sea level 4–8 m higher than in the Present during MIS 5.5^5^. Moreover, the occurrence of "Senegalese fauna", including the most characteristic gastropod species *Strombus bubonius,* in these beach deposits was considered distinctive of the MIS 5.5 highstand^6,7^.

A more recent study^8^ proposed a remarkably different scenario for the northern-central coast of Latium. This sector is characterized by the presence of three marine terraces at elevations of ~ 35, ~ 24, and ~ 12 m a.s.l. Compared to previous estimations for the Mediterranean Sea within the 125–80 ka interval (including three highstands marked as 5.5, 5.3 and 5.1), the sector reveals significantly different amplitudes of the sea-level oscillations. Successive studies^9,10^ questioned the attribution to MIS 5.5 of the paleo-coastline occurring at ca. 8–9 m in the coastal reach between the Circeo promontory and the Gaeta gulf (Fig. 1b). Based on the occurrence of the same set of terraces in the south of Anzio, these authors suggested that this coastal sector also underwent the uplift found in the coast between Anzio and Argentario promontories, meaning that the MIS 5.5 paleo-coastline should have been at ~ 35 m a.s.l.

Establishing the maximum amplitude of the sea level during the two sub-stages following the penultimate glacial maximum is very relevant to climate studies, particularly to the commonly accepted notion that the benthic d18O record provides a very reliable estimation of the global ice volumes (e.g.,^11,12^). Recent work at the Balearic Island suggested a similar sea level during MIS 5.5 and MIS 5.1 in the Mediterranean Sea^13,14^, prompting a debate about the reliability of U-Th dating methods and the Glacial Isostatic Adjustment (GIA) effects^15^. However, a recent compilation of coral reef sea-level benchmarks^16^ outlined that both MIS 5.3 and MIS 5.1 display significant offsets with respect to the estimated global sea levels^12^, with elevation ranging − 25–0 m. Such variations have been attributed to unforeseen tectonic or Glacial Isostatic Adjustment (GIA) effects. This work renewed a well-known problem concerning the 80 ka MIS 5.1 sea level, which was already discussed by^17^. Finally, Marra et al.^10^ have provided geochronologic constraints to the MIS 5.5 marine terrace occurring at 31–40 m along the central Tyrrhenian Sea coast between the Argentario and Circeo promontories. They have shown that, assuming a constant tectonic uplift of 0.24 mm/year during the last 125 ka, the sea-level associated with this terrace and with two lower terraces at 24–28 m and 11–17 m were + 6 m (pre-assigned), ca. + 1 m, and ca. − 7 m for MIS 5.5, MIS 5.3 and MIS 5.1, respectively.

## Methods

In this paper, we present a detailed original analysis of the stratigraphic records of Grotta Guattari and Grotta dei Moscerini by examining new investigations performed at Grotta Guattari as well as materials recovered at Grotta dei Moscerini during past archaeological excavations. By cleaning and deepening previously excavated archaeological sections at Grotta Guattari, we provide a detailed description of the stratigraphic features (with significant revision of published stratigraphies^2,18^) aimed at establishing the paleoenvironmental characters of the sediments and their implications on the past sea level.

Moreover, we recovered unpublished stratigraphic notes performed by Aldo Segre during the 1949 excavations at Grotta dei Moscerini. Since the entrance of this cave is now hampered by a landslide which occurred as a result of coastal road construction, these notes represent the only detailed description of the complex stratigraphic features of the cave’s sedimentary filling. These notes, combined with the observations and petrographic/geochemical analyses on a set of pumice clasts (part of the sedimentary deposits of Grotta dei Moscerini stored) and other archaeological materials at the IsIPU repository in Anagni, allowed us to closely investigate the lithostratigraphic and paleogeographic implications of the sedimentary filling of this cave as well. Results of the petrographic and geochemical analyses, promoted by INGV and La Sapienza University, have been partially shared with^4^. Compared to previous work, in which only the TAS compositions of this pumice were discussed, we provide geochemical comparison with the distal tephrostratigraphic record using new bivariate diagrams. This allows for an excellent discrimination of their source area and a precise estimation of the eruption ages.

All the new data collected for this work allowed us to investigate the relationships between sea-level oscillations and deposition of the sedimentary fillings of these caves, providing new insights on the absolute sea level during MIS 5.3 through MIS 5.1.

Throughout the paper we refer to different dating methods used in previous literature to provide geochronologic constraints to the sea-level markers along the Latium coast, including ^40^Ar/^39^Ar, ESR-U series (both on mollusk shell and tooth enamel), aminoacid dating, and biochronology. All the ages provided through these methods have, at different extents, associated uncertainties and analytical problems, and thus should be regarded with caution. An in depth discussion of these ages and their reliability is provided in Supplementary Material 1.

### Permissions and materials

Authorization for the analysis and photographs of the pumice recovered during excavations at Grotta dei Moscerini has been granted to the Istituto Nazionale di Geofisica e Vulcanologia (INGV) by the Istituto Italiano di Paleontologia Umana (IsIPU) Director, Stefano Grimaldi. The stratigraphic study of Grotta Guattari has been possible thanks to the cleaning of the sections previously excavated and to the collection of new geologic samples for petrographic and geochronologic analyses, performed under the concession by 'Soprintendenza Archeologia, Belle Arti e Paesaggio del Lazio'.

## Results

### Extending the marine terraces south of Anzio promontory

A suite of paleo-surfaces was reconstructed by^10^ through a combined geomorphological/statistical study in a 200 km-long coastal sector of the central Tyrrhenian Sea between Argentario and Circeo promontories. Results of this study show:

1. three marine terraces homogeneously extending through the coast of central Italy, which are identified by paleo-surfaces ranging 31–40 m, 24–28 m, and 11–17 m a.s.l.;
2. nearshore deposits associated with these terraces providing paleo sea-level elevations of ~ 35 m, ~ 23 m, and ~ 12 m a.s.l.;

Available geochronologic and biochronologic constraints, within the limits of the methods and associated uncertainties (see Suppl. Mat. 1), provide correlation for the highest sea level of ~ 35 m with MIS 5.5 and for the lowest ~ 12 m sea level with MIS 5.1, allowing to assign the intermediate ~ 23 m sea level to MIS 5.3.

In particular, the suite of three terraces was extended between Anzio and Circeo promontories (Fig. 1b) by combining a novel geomorphologic study with the physiographic study conducted by^19^. The latter study had already recognized two marine terraces corresponding to the Latina and Minturno complexes, and outlined the occurrence of a third, upper terrace: the Campoverde complex^9,10^ (Fig. 1b). A fourth marine terrace at 2–3 m a.s.l. was identified by^19^: the Borgo Ermada complex. The chronology of this terrace and the relationships with the sea level were recently analyzed proposing an *ante-quem* age of 41–36 ka and a mainly regressive character for it^20^.

In this work we provide new evidence for the presence of this suite of four terraces also at the Circeo promontory, by rescuing old papers published only in local journals, which were disregarded by more recent literature. Indeed, the occurrence of these marine terraces and the associated plaeo-sea levels as provided by the combined morpho-stratigraphic record of^19^ and^9,10^ finds a straightforward match with early observations at Monte Circeo, in the surroundings of Grotta Guattari, reported by^21^. These authors identified three coastlines (SL_1_, SL_2_, SL_3_) and their related terraces (T_1_, T_2_, T_3_), as shown in insets of Fig. 1c, d, based on the identification of three distinct "beach deposits" (all characterized by the presence of "Senegalese fauna") and three related orders of "morphologic surfaces of marine origin". Moreover, a fourth, more subdued, higher coastline (SL_0_) was identified at 35–45 m in the area of Grotta delle Capre and Grotta del Fossellone^22,23^. Elevations of the three lower coastlines at 2–3 m, 8–10 m, and 20–25 m (SL1, SL2, SL3 in Fig. 1c, d) match those of the three marine terraces of Borgo Ermada, Minturno and Latina complexes described by^19^, as well as the elevations (within the associated errors) of the two sea-level associated with the 11–17 m and with the 20–28 m terraces (i.e., ~ 12 m and ~ 23 m) in^10^ (Fig. 1b). The coastline of 35–45 m finds its previously unrecognized morpho-stratigraphic expression in the 31–40 m terrace of the Campoverde complex and the associated sea level of ~ 35 m, introduced by^9,10^ (Fig. 1b).

Additionally, it is remarkable that^21^ highlighted the good match among the three lower coastlines identified at Circeo and the three "*Strombus* raised beaches" occurring at 2–3 m, 10–15 m, and 18–25 m, recognized in northern Latium by^24^. Although unexpectedly discarded by later literature, this widespread occurrence of a suite of three marine terraces at homogeneous elevations throughout the Latium coast has been confirmed in the recent geomorphological, stratigraphic and geochronologic study which reconstructed a flight of three terraces^8–10^. In this study, the lowest two terraces, characterized by the presence of *S. bubonius*, corresponded to the two upper "*Strombus* raised beaches" of the Authors. In contrast, the highest terrace of 31–40 m highlighted in the recent reconstruction was only discontinuously recognized between the Argentario and Anzio promontories in previous studies (e.g.: Cava Tacconi^25^; Monna Felice and Casale di Statua^26^) which had attributed it to MIS 5.5 but interpreted it as the same, tectonically uplifted, terrace occurring elsewhere at around 2–3 m, 10–12 m, and 24–27 m.

Regardless of their ages, this set of four terraces and related sea levels at ca. 35, 23, 12, and 2 m appears as a homogeneous geomorphologic/paleogeographic feature of the Latium coast, extending from Monte Argentario to Circeo promontories at least^10^. Indeed, this uniform morpho-structural setting speaks for a homogeneous tectonic regime affecting the Tyrrhenian Sea margin of central Italy during the last 250 ka, consistent with the fact of being part of the same geodynamic domain represented by the back-arc extensional region. This sector is located at the rear of the northern Apennines orogenic belt, extending from Tuscany to southern Latium, where the southern boundary is represented by the Olevano-Antrodoco Line (the outermost thrust front of the northern Apennine's arc^27^; Fig. 1a).

However, the higher 31–40 m terrace and its sedimentary record of nearshore deposits is geochronologically tightly constrained within MIS 5.5 by a ^40^Ar/^39^Ar age of 131 ± 2 ka at Cava Rinaldi near Rome^28^ and by a maximum age of 184 ± 9 ka in Rimessa di Montalto near the Argentario^10^. By contrast, in south of Anzio, it has only combined biochronologic and geochronologic constrains in Campoverde, suggesting correlation with MIS 5.5^10^ (Fig. 1a; see Suppl. Mat. 1). On the other hand, consistent ESR/U-series ages on *Glycimeris* shell and tooth enamel provide ages of 74 ± 7, 77 ± 9^29,30^, and 79 ± 7 ka^10^ for the deposits forming the 11–17 m terrace between Anzio and Grotta dei Moscerini, in good agreement with the correlation of this flight of progressively lower terraces at 31–40, 20–28, and 11–17 m with MIS 5.5, 5.3 and 5.1, respectively (Fig. 1e). It must be noted that Marra et al.^10^ have observed that the sea-level markers occurring at Grotta Guattari and Grotta dei Moscerini are also compatible with an age within MIS 5.5 when the absence of uplift in this coastal reach is assumed and the similar maximum sea-level during the following highstands of MIS 5.3 and MIS 5.1 (Fig. 1e) is considered. This hypothesis is consistent with data from the coastal sector where the MIS 5.5 sea-level markers are located around 35 m a.s.l., which assuming a constant tectonic uplift during the last 125 ka and a maximum sea level 6 m above Present for MIS 5.5 highstand, provide an estimation of ca. + 1 and ca. − 7 m a.s.l. for MIS 5.3 and MIS 5.1.

### Grotta dei Moscerini

**Figure 2:**
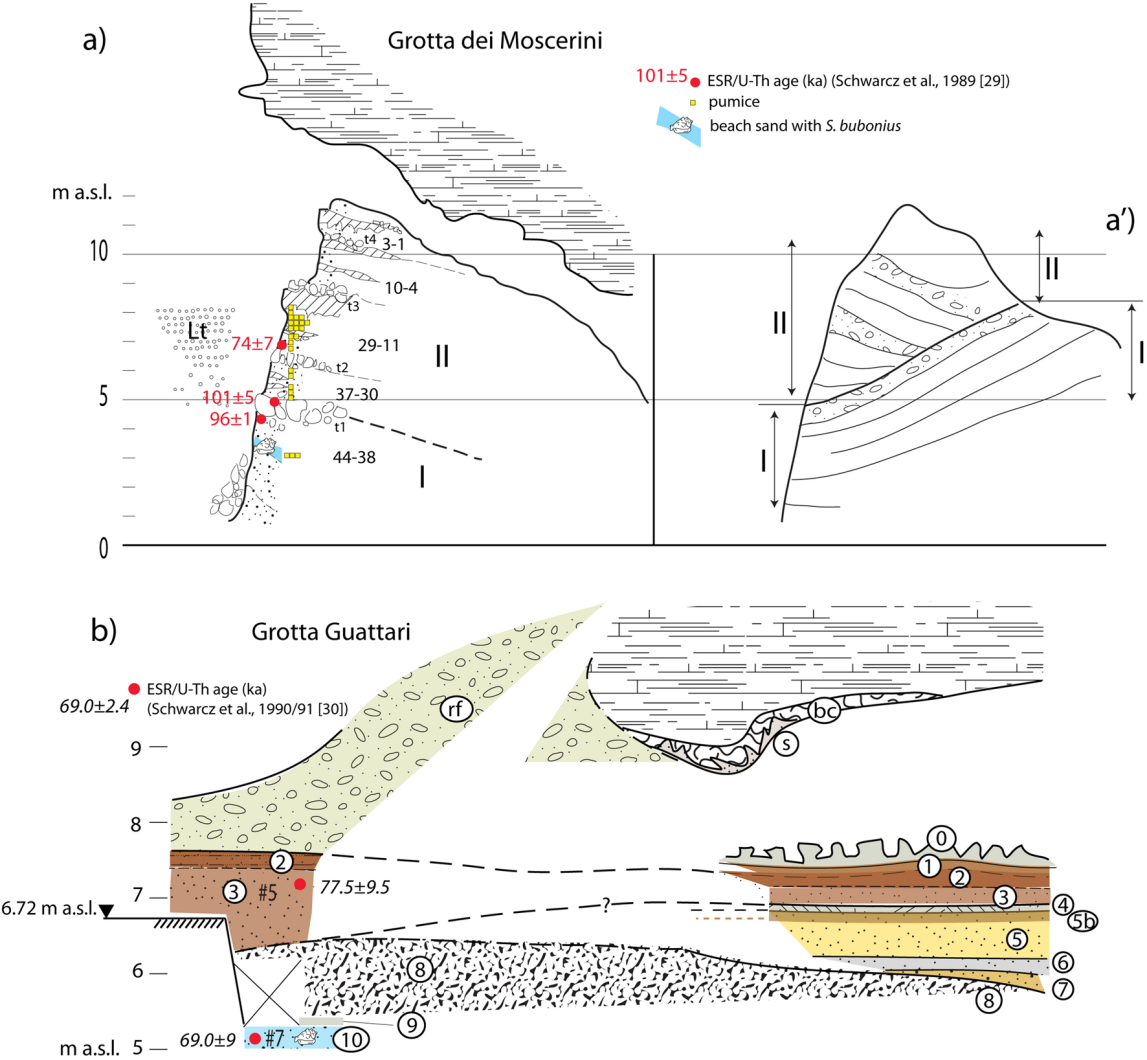
(**a**, **a′**) Stratigraphic sketches of Grotta dei Moscerini redrawn by F. Marra based on unpublished field drawings by A. Segre stored at the IsIPU Repository (Anagni, FR, Italy). Legend: Lt, lithodomus burrows; t, talus layer; layer numbering 1–44 according to unpublished stratigraphy by Aldo Segre synthesized in^31^. (**b**) Stratigraphic sketch of Grotta Guattari based on new field work performed for the present study; drawing by F. Marra. Legend: *rf*—rockfall; bc—cemented conglomerate with mollusk shell fragments; s—grey, femic sand filling the fractures in bc; 0—encrusted rockfall; 1—earthy deposits with small rock fragments; 2—dark brown to dark reddish (outer section), pedogenized sand; 3—brown to reddish (outer section) medium sand; 4—cm-thick carbonatic crust; 5b—slightly pedogenized layer; 5—dark yellow sand, with carbonatic concretions and rare, minute, well rounded gravel (outer section); 6—grey sand; 7—reddened coarse sand with rare, well rounded pebbles; 8—cemented, rounded carbonatic blocks in coarse sand matrix; 9—dark brown, loose sandy deposit; 10—biodetritic sandy conglomerate. Black triangle—GPS measured elevation. See text for explanations.

The stratigraphic record of Grotta dei Moscerini was described in detail by Aldo Segre (a synthesis is reported in^31^), who identified 44, cm- to dm-thick, distinct layers (Fig. 2a). For a detailed archaeological interpretation of these layers see^4^.

We have recovered the original handwritten notes by A. Segre stored at the IsIPU repository and by combining them with the ESR age constraints provided by^29^, we have reconstructed the following stratigraphic-sedimentologic features.

The filling of Moscerini cave consists of a basal beach deposit with mollusk shells (including *Strombus bubonius*), followed by a circa 8 m thick complex succession of continental deposits, including alternating sandy and clay layers, burned or carbon-rich layers, and stalagmitic crusts. Four major layers constituted of large (dm- to m-sized) fallen blocks of calcareous rock (talus) are intercalated at different elevations in the sedimentary succession (t1-4 in Fig. 2a). These are interpreted by Segre as crioclastic conglomerate (i.e. originated by congealment of the water filling the rock fractures), possibly corresponding to the occurrence of a glacial period. The most significant characteristic of the sedimentary record of the cave, however, is the diffused occurrence of Mousterian tools made on the shell of a marine clam (*Callista chione*)^3,4^. A series of lithophaga burrows (Lt in Fig. 2a) was also described to occur on the outer, northern wall of the cave, approximately between 5 and 8 m a.s.l. We remark that a precise geodetic survey was not conducted at Grotta dei Moscerini, therefore all the elevations reported in the present paper are estimated based on the referred elevation of 3 m a.s.l. for the base of the stratigraphic section and on its thickness as reported in^31^.

**Figure 3:**
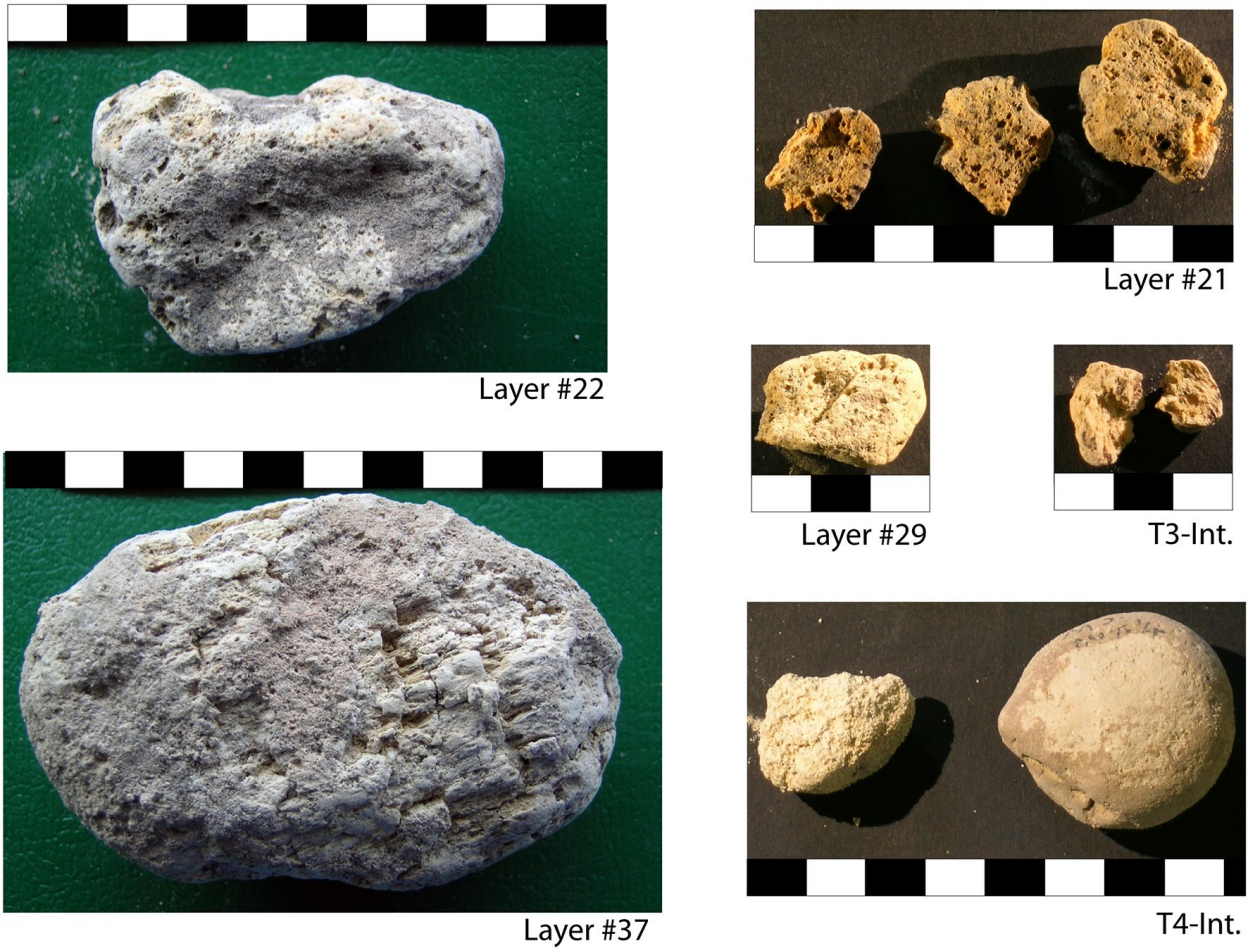
Photographs of pumice samples from Grotta dei Moscerini (by concession of IsIPU).

A widespread occurrence of rounded pumice clasts, reported in the unpublished stratigraphy by Segre and recently discussed in^4^, has been outlined in the present study through the re-examination of the materials recovered during the original excavations. Pumice clasts from ca. 1 cm up to ca. 10 cm in diameter have been found in the materials labeled as pertaining to layers 37, 30, 29, and continuously from 26 to 19, with a maximum concentration (31 specimen) in layers 23–22–21 (Fig. 2a). Moreover, abundant pumice occurrence is reported by Segre in layer 44 and, at lesser extent, in layers 35–36. Finally, several pumice clasts have been recovered in the interior cuts 1, 3, and 4 during archaeological excavations, for which the stratigraphic position is undetermined. Three pumice clasts from layers 37, 22, 21 and two from interior cuts 3 and 4, a have been selected for petrographic and geochemical (SEM) analysis (Fig. 3). Moreover, pumice samples from layer 37 and 22 have been selected for ^40^Ar/^39^Ar dating which are in progress within the ongoing "Guattari project", aimed at providing new geochronologic constraints to the time of frequentation of the coastal caves of southern Latium and assessing the timing of the galacio-eustatic fluctuations during MIS 5, precisely.

Figure 2 shows the longitudinal (a) and transversal (a′) cross-sections of the cave filling described by^31^ in which a marked discordance separates a lower, progradational wedge (I) from an upper, retrogradational wedge (II). These sedimentary wedges correspond to two main transgressive cycles (I and II in Fig. 2a), accounting for two coastlines approaching the cave entrance in different epochs. Two groups of ESR/U-Th ages performed on tooth enamels recovered in the sedimentary filling of the cave^30^, providing average ages of 96 ± 1/101 ± 5 ka and 74 ± 7 ka (Table 1b in Suppl. Mat. 1), suggest correlation of the corresponding sea-level markers with MIS 5.3 and 5.1 highstands, respectively. As mentioned before, accuracy of these ages is questionable (see Suppl. Mat. 1); however, current literature on the Neanderthal frequentation of the cave refers to them in lack of any other available geochronologic constraints (^4^, and references therein). In the present work we compare these ages with the chronology of the Ischia and early Phlegraean Fields eruptive activity, through geochemical fingerprinting of the pumice contained in the sedimentary filling of Grotta dei Moscerini.

**Table 1:** EMP glass analysis.

Pumice sample	37	37	T4	T4	T4	T4	T4	T4	T4	T3	T3	T3	T3
SiO_2_	60.94	61.58	60.41	57.27	62.12	61.56	56.53	61.18	60.26	61.16	60.92	60.96	62.60
TiO_2_	0.62	0.61	0.61	0.57	0.63	0.61	0.51	0.61	0.60	0.40	0.40	0.38	0.31
Al_2_O_3_	17.24	17.63	17.46	16.78	17.86	17.45	16.10	17.63	17.12	18.48	18.53	18.38	18.98
MgO	0.29	0.29	0.38	0.31	0.32	0.32	0.41	0.33	0.33	0.60	0.57	0.57	0.53
CaO	0.96	0.95	0.93	0.86	0.93	0.95	0.92	1.00	0.90	2.50	2.50	2.41	2.57
MnO	0.34	0.30	0.37	0.31	0.40	0.42	0.31	0.38	0.36	0.15	0.15	0.15	0.19
FeO	2.74	2.81	2.74	2.61	2.86	3.02	2.66	2.87	2.72	3.23	3.20	3.01	3.13
Na_2_O	7.93	7.82	7.71	7.46	7.84	7.76	6.68	7.95	7.90	3.29	3.16	2.60	2.40
K_2_O	5.99	5.92	5.97	5.91	6.29	6.11	5.48	6.22	5.96	8.86	8.55	8.54	8.24
P_2_O_5_	0.03	0.07	0.03	0.07	0.00	0.05	0.05	0.03	0.00	0.06	0.15	0.12	0.09
SO_3_	0.11	0.03	0.05	0.09	0.00	0.04	0.04	0.02	0.03	0.12	0.11	0.12	0.14
F	0.31	0.45	0.44	0.24	0.49	0.54	0.31	0.19	0.56	0.08	0.18	0.27	0.07
Cl	0.73	0.71	0.79	0.68	0.75	0.76	0.83	0.78	0.71	0.48	0.46	0.41	0.43
Total	98.24	99.16	97.91	93.15	100.49	99.59	90.83	99.18	97.44	99.39	98.88	97.94	99.66
SiO_2_*	60.94	61.58	62.53	62.15	62.59	62.66	63.06	62.30	62.67	61.16	60.92	60.96	62.60
K_2_O + Na_2_O*	14.34	14.02	14.16	14.51	14.24	14.12	13.56	14.43	14.41	12.31	11.93	11.47	10.74
*Recalculated at 100 on dry basis													
K_2_O/Na_2_O	0.76	0.76	0.77	0.79	0.80	0.79	0.82	0.78	0.75	2.69	2.71	3.28	3.44

### Grotta Guattari

A stratigraphy similar to that of Grotta dei Moscerini has been described at Grotta Guattari (Fig. 2b) where, according to^29^ “the sea periodically entered the cave during isotope stages 5e–5a” (i.e., 5.5–5.1). A coarse beach sand deposit (layer #7, Fig. 2b) with mollusk shells occurring at 5.2 m above present sea level (geodetic point elevation measured for the present study) has been correlated by these authors with isotope stage 5a, based on one ESR/U-Th age of 77.5 ± 9.5 ka yielded by a *Bos* tooth from an overlying continental sand deposit (layer #5). This age is remarkably similar to that of 74 ± 7 ka constraining the second transgressive cycle at Grotta dei Moscerini, also performed on a tooth recovered within a sandy layer in the upper depositional cycle. However, unlike in Grotta dei Moscerini where the sand deposit is part of a different depositional cycle than the underlying beach deposit, no evident unconformity was described to occur in the sedimentary succession at Grotta Guattari, where another ESR/U-Th age on a tooth from layer #7 yielded 69 ± 9 ka^29^. Although the authors have argued about the stratigraphic inconsistency of this apparently younger age, we remark that the two ages overlap each other within the associated analytical errors and should be considered indistinguishable.

**Figure 4:**
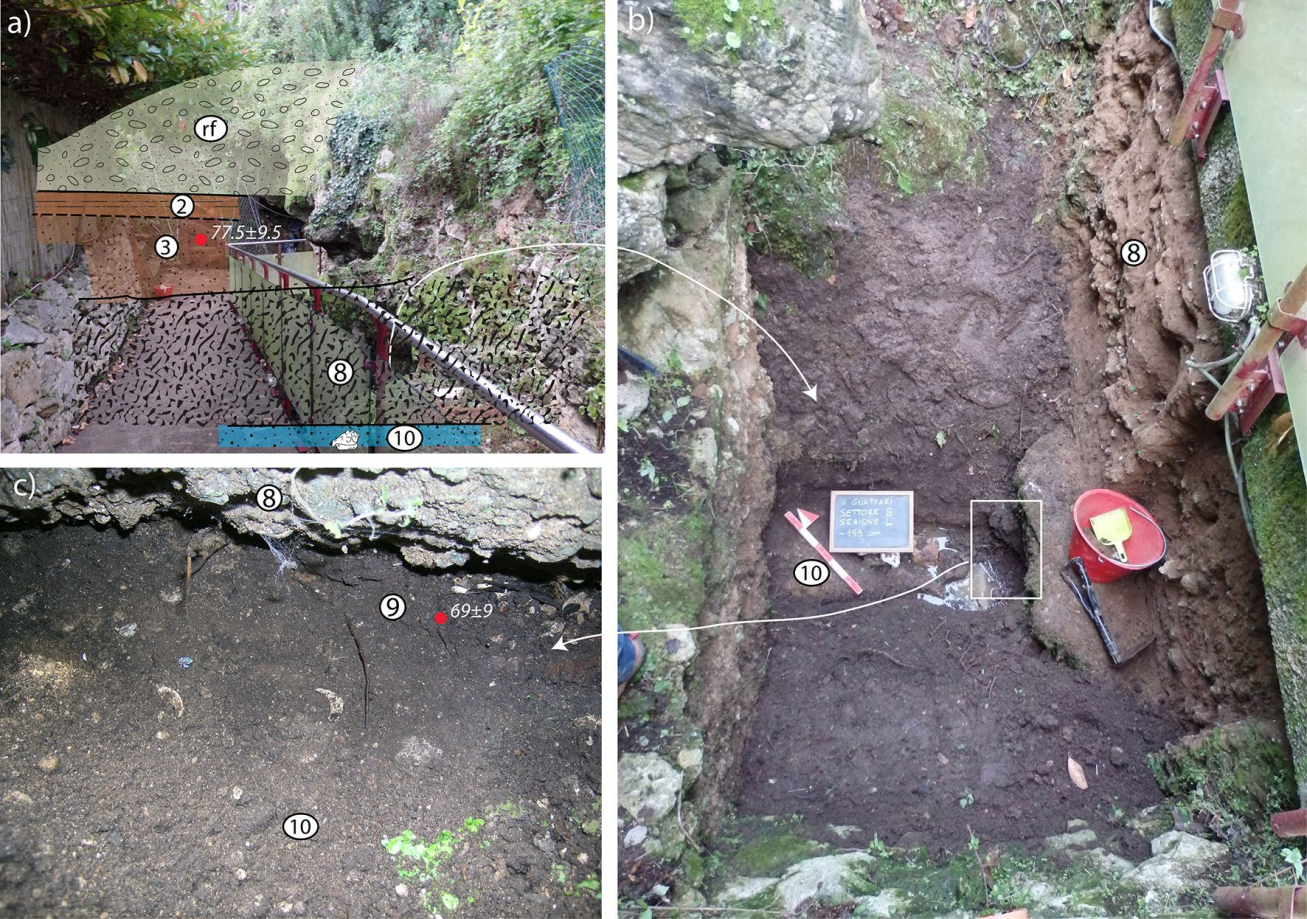
Photographs of the exterior of Grotta Guattari showing detail of the stratigraphy exposed by cleaning of the archaeological sections. Photos taken by F. Marra and published under the permission by Soprintendenza Archeologia, Belle Arti e Paesaggio del Lazio. Legend (as in Fig. 2b): 10—biodetric sandy conglomerate; 9—brown, earthy sand; 8—cemented, rounded carbonatic blocks in coarse sand matrix; 3—dark yellow sand with carbonatic concretions and rare, minute, well rounded gravel; 2—reddish-brown, pedogenized sand; rf—rockfall. See text for comments.

During the new investigations that we performed at Grotta Guattari one task was specifically addressed at clarifying the stratigraphy of its sedimentary filling. Our reconstruction, comprising 10 stratigraphic units (SU) below the cave floor represented by a thick carbonate incrustation conglobating a crioclastic conglomerate and numerous vertebrate bones (SU 0), is summarized in Fig. 2b. A notable difference emerged with respect to both the stratigraphy described by^18^ and that reported by^2^ and adopted in^29^. All these previous authors reported a continuous stratigraphy with from bottom to top:

1. biodetritic conglomerate, grey sandy soil with shell fragments (layer #7 and #6 according to^2^); these correspond to layer 8: beach sand with shell fragments, slightly pedogenized in the upper part, according to^18^. We have reported these deposits as 10 and 9, respectively, in Figs. 2b and 4.
2. sandy soil containing pebbles, sandy soil with limestone concretions, compact brown earth, loose brown earth, earth with small limestone blocks (layers #5, #4, #3, #2, #1 by^2^). These units are summarized as reddened sand dune passing upward to a sandy paleosoil (layers 9–9′) by^18^). Layers 9–9′ seem also to correspond to those cropping out in front of the cave entrance that^29^ reports as layer #5 according to Taschini's^2^ stratigraphy, in which sample for ESR dating was collected. Similarly to Taschini^2^, we have recognized several SUs in this interval (7 through 1 in Figs. 2b and 4).

Moreover, in contrast with what reported above, we have ascertained that the lowest layer (SU 10 in Figs. 2b and 4), a cemented biodetritic sand and fine gravel with abundant, often intact mollusk shells (mostly *Glycimeris*), is separated from the overlying sand layer by a circa 150 cm thick cemented layer (SU 8 in Figs. 2b and 4) made of decimeter-sized calcareous, rounded blocks within a brown, altered sand and clay matrix, sealed by a cm-thick carbonatic crust. Although previous authors did not report it, the "beach" layer underlies the cemented calcareous blocks at the base of the front wall of the cave. We could easily detect it because of ca. 20 cm thick layer of loose, brown, earthy sand separating the top of the biodetritic stratum from the base of the overlying conglomerate (SU 9 in Fig. 4c). Moreover, we have cleaned the level at the base of the section exposed in front of the cave entrance and, ca. 60 cm below the ground level, we have found the top of the large blocks conglomerate underlying the continental sand deposit (Fig. 4a).

In addition to the different stratigraphic setting with respect previous literature descriptions, we have found no evidence neither for the lithophaga burrows reported in the graphic sketch of the cave by^21^, nor for a tidal notch reported to occur at 9.2 ± 0.5 m by^26^. In contrast, we have observed a very well preserved tidal notch described by^26^ to occur at 9.6 m (± 0.5) in the nearby Grotta delle Capre, where a thick band of lithophaga burrows also occurs at lower elevation.

### Petrographic and geochemical analyses of Moscerini cave pumice

At the optical microscope, the investigated pumice clasts (Fig. 3) display vesicular texture and appear poorly porphiritic. Samples T4 and #21 are practically aphiric, while samples #37, #22, and T3 are characterized by total phenocryst content ≤ 2 vol%.

The dominant phenocrystic phase, generally present as glomerocrysts, is constituted by Feldspars, along with minor black mica and clinopyroxene. Accessory phases are represented by opaque oxide minerals, apatite and sphen. The mineral assemblages vary according to the chemical composition of the rock.

**Figure 5:**
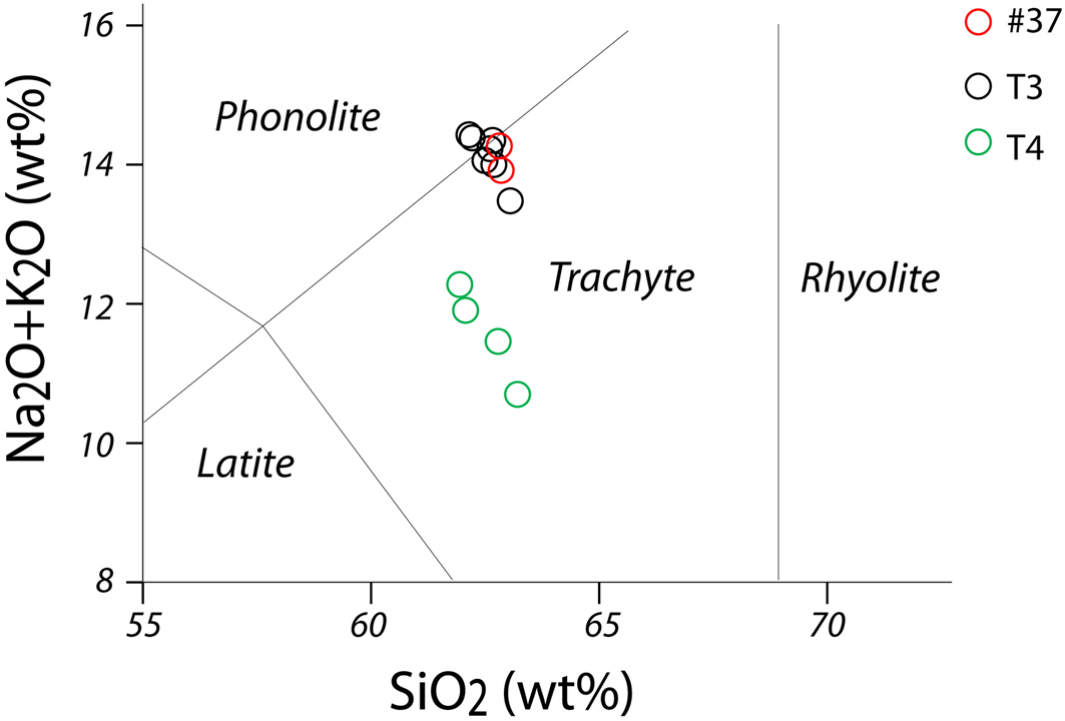
TAS diagram of analyzed pumice samples.

Sample #37 has phono-trachytic composition in the TAS diagram of Fig. 5 and is characterized by dominant alkali-feldspar, minor biotite and accessory phases. Sample T3 is trachytic, with dominant plagioclase, rare clinopyroxene, and minor content of black mica and accessory phases. Both samples are characterized by a glassy groundmass with rare microlites, which are not optically resolvable.

**Figure 6:**
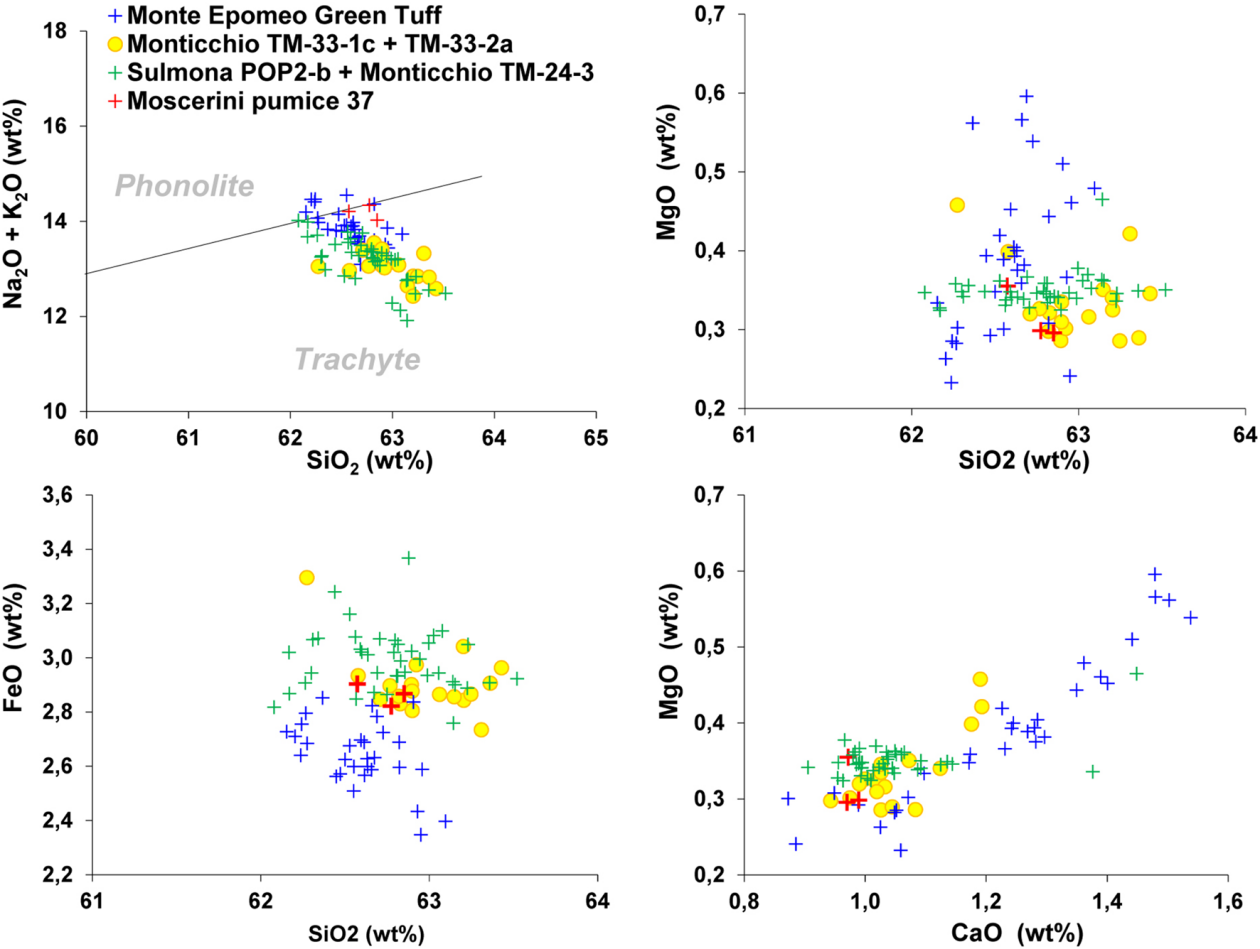
Total alkali versus silica classification diagram and representative bi-plots for the Moscerini pumice #37 and of some potential correlative tephra from Ischia Island activity. Data source TM-33-1c, TM-32-2a and TM-24–3 (glass-WDS)^50^; POP2b (glass-WDS)^32^; Monte Epomeo Green Tuff (glass-WDS)^51^.

Pumice samples #37 and T4 have an alkali-rich composition at the border between the phonolitic and trachytic field in the TAS diagram (Fig. 5) which, combined with a K_2_O/Na_2_O < 1 (Table 1) and their mineralogical assemblage, matches the typical composition of the products of Ischia Island. For comparison, we considered all the dozens of Ischia tephra occurring in the rich Monticchio lacustrine basin record between ca. 56 and 133 ka (Epomeo Green Tuff and Y-7 tephra)^32,33^ (and references therein). Though Ischia tephra share a similar trachyte composition, almost indistinguishable in TAS diagram, they can be better discriminated using other bivariate diagrams (Fig. 6). In particular, we found a good match of Moscerini pumice #37 with tephra TM-33-1c/TM-33-2a dated to 118.2 ± 5.9 and 116.10 ± 5.8 ka, respectively, or with the tephra TM-24–3, also fund in Sulmona basin (POP2b,^32^) and dated to ~ 104.0^33^. These distal tephra are correlated with two eruptive units from Ischia: Punta Imperatore and Mt. Sant'Angelo^34,35^.

Therefore, a time span ranging 118–104 ka can be assumed for pumice #37, consistent with ESR age constraints ranging 101 ± 5–96 ± 1 ka for the layers #38 and #39 of the sedimentary filling of Grotta dei Moscerini.

Pumice sample T3 displays trachytic composition in the TAS diagram, typical of the pre-Campanian Ignimbrite Phlegrean Fields products. Also, in this case, this eruptive period is poorly known from a geochronologic point of view; however, regional tephrostratigraphic data constrain it between 110 and 40 ka^36,37^, providing further support to the chronologic framework of Grotta dei Moscerini achieved from ESR dating.

However, all the analyzed pumice clast are strongly weathered and reworked, revealing sea-water transport and successive gathering on the beach. Therefore, the pumice fragments could have spent a significant portion of time in the ocean system prior to being deposited in sediments. Thus, their eruption age should be considered as *post-quem terminus* for emplacement.

## Discussion

### Correlation of the sea-level markers

The two beach sand deposits of Grotta Guattari and Grotta dei Moscerini are associated with a sea level at 8–9 m a.s.l. which correlates that occurring progressively at slightly higher elevation in Borgo Sabotino (10 m,^26^), associated with the marine terrace of the Minturno Complex^9,11^, and in Quadrato (~ 12 m), where it is dated by ESR/U-Th age on *Glycimeris* at 79 ± 7 ka^10^ (Fig. 1e).

A rather consistent picture emerges from cross-section in Fig. 1e, based on the available geometric and geochronologic constraints on the sea-level markers along the southern Latium coast between Anzio and Gaeta promontories. Apparently, these data support the attribution to MIS 5.1 for the 8–10 m paleo-sea level, with respect to the previous interpretation of a MIS 5.5 age essentially based on the occurrence of Senegalese fauna and on aminoacid dating of several mollusk shells^38^. However, as suggested by^10^, *S. bubonius* cannot be considered a distinctive marker of MIS 5.5 in this region, since in the coastal sector between the Argentario and the Tiber mouth it is widespread, possibly as reworked occurrence, in the lowest terrace at 11–15 m, and also occurs in the intermediate terrace of 24–27 m, while it is never found on the uppermost 31–40 terrace which is geochronologically constrained by a ^40^Ar/^39^Ar age of 129 ± 2 ka within MIS 5.5^28^. This has been suggested to be due to the deeper erosion affecting the beach facies of this oldest terrace, for which only the more inland lagoon and coastal plain deposits are preserved^10^. The widespread occurrence of reworked mollusk shells in the two lower terraces also may account for a few amino acid ratios considered distinctive of MIS 5.5 yielded by four mollusk shells collected at different locations within the deposits associated with these sea-level markers. Indeed, we remark that only at four sites the dated mollusk specimens were collected outside of the tectonically active^39^ Pontina and Fondi plains (Fig. 1b), where all the other dated specimens^38^ were collected and for which their original elevation is undetermined and are unfit to provide reliable indications. In contrast, a MIS 5.5 age for the *Strombus* collected 10 m a.s.l. in Borgo Sabotino^6^ is conflicting with the ESR/U-Th age of 79 ± 7 ka yielded by the *Glycimeris* shell collected at the same elevation at the nearby Quadrato site, suggesting that the former is a reworked specimen, as the widespread reworking affecting the thanatocoenosis of this coastal terrace outlined in^10^ supports. Remarkably, two distinct amino-acid ratios were reported for two shell specimens collected in Quadrato by^6^, who already argued on the reworked features of the oldest specimen, as well as on the reworked features of the abundant *Strombus* specimens collect at this site by^40^.

The considerations above apply to the coastal reach immediately south of Anzio, where the four terraces reconstructed in^10^ have marked expression (Fig. 1b), while an evident discontinuity of the two upper terraces characterizes the intermediate coastal reach between Anzio and Circeo. However, report of the tidal notch at ca. 20 m and of the subdued terraced surface at 40–50 m in^21–23^ suggests tectonic uniformity throughout this area. In contrast, the southernmost coast through Grotta dei Moscerini and Gaeta may represent a tectonically stable sector, consistent with the lack of evident marine terraces and a markedly lower elevation (~ 5 m a.s.l.) of the tidal notches occurring at three sites near Gaeta^26^.

With all these elements in mind, in the following sections we attempt at reconstructing possible scenarios for the sea-level oscillations, accounting for the different interpretations about the age of the sea-level markers occurring at Grotta dei Moscerini and Grotta Guattari.

### Reconstruction of the sea-level history

A methodological premise on the identification of the sea-level markers is fundamental when dealing with the sedimentary record preserved within a coastal cave. Indeed, unlike for a coastal plain where a morpho-stratigraphic complex represented by a sedimentary succession and a terraced surface allow for identification of the paleo-coastline and the corresponding maximum sea-level (e.g.,^41,42^), such elements cannot be recognized in a steep coastal reef, such as that of the Circeo promontory. Here, caves are sedimentary traps, in which beach deposits are not associated with a terrace, preventing from telling whether they were deposited during the highstand or during any time pertaining to the ingressive or the regressive phase. In other words, beach deposits within a cave are not indicative of the highstand maximum sea level, which can be identified only by tidal notches and by lithofaga burrows (e.g.,^5^). In contrast, a transgressive succession comprising near-shore deposits (beach, as well as lagoon facies) overlain by a dune facies at the top of a sedimentary complex defining a marine/coastal terrace provides a paleo-sea level marker with good approximation, in the order of few meters^10,19,42^.

### Grotta dei Moscerini

The set of sea-level markers represented by the basal beach sand layer and the lithophaga burrows occurring at ~ 3 m and between ~ 5 and ~ 8 m a.s.l., respectively, is clearly associated with the lower transgressive cycle, as also suggested by the presence of lithophaga burrows on some of the fallen calcareous blocks within the crioclastic layer t1. However, if we correlate the first depositional cycle with MIS 5.3, as suggested by the ESR-U/Th ages spanning 101 ± 5–96 ± 1 ka, the occurrence of a 100 ka paleo-coastline at ca. 8 m would be conflicting with the elevation of the corresponding marine terrace in the coast between the Argentario and Anzio promontories and the estimated paleo-sea level at ca. 23 m a.s.l. Therefore, the most likely hypothesis is that the first depositional cycle correlates the time span encompassing MIS 5.5 highstand and the following regressive phase towards MIS 5.4 lowstand (ca. 110 ka), consistent with maximum age attributed to pumice #37 through the correlation with the 118–104 ka eruptive units of Ischia.

We remark that pumice clasts occurrence seems markedly correlated with proximity of the coastline, as they occur within the beach deposit of layer #44, and in the layers #37 through #19, which are in the same elevation range as the lithophaga burrows (Fig. 2a). Indeed, such well-rounded pumice fragments transported by the sea currents are commonly gathered by wave action on the beaches of Latium, and may also be incorporated in the wind-blown, backbeach deposits. In the case of Grotta dei Moscerini, however, the great abundance of pumice within the sediment accounts for a large input, which is suggestive of their sin-eruptive origin. Notably, ESR age interval for the sedimentary succession overlaps the early eruption phases occurred at Ischia and at the Phlegraean Fields, supporting reliability, within the associated uncertainties, of the ESR age performed by^30^. Therefore, the large amount of pumice in the portion of the cave filling dated at 74 ± 7 ka evidences the backbeach origin of the deposit, outlining a sea level not much lower than 8 m a.s.l. during MIS 5.1. Such inference is consistent with hypothesis already formulated in^10^ for a stable tectonic regime in this portion of the coast, accounting for similar maximum sea levels during MIS 5.5 though MIS 5.1 highstands. Based on the elements described above, a possible sea-level history at Grotta dei Moscerini is reconstructed hereby.

### Cycle I

**Figure 7:**
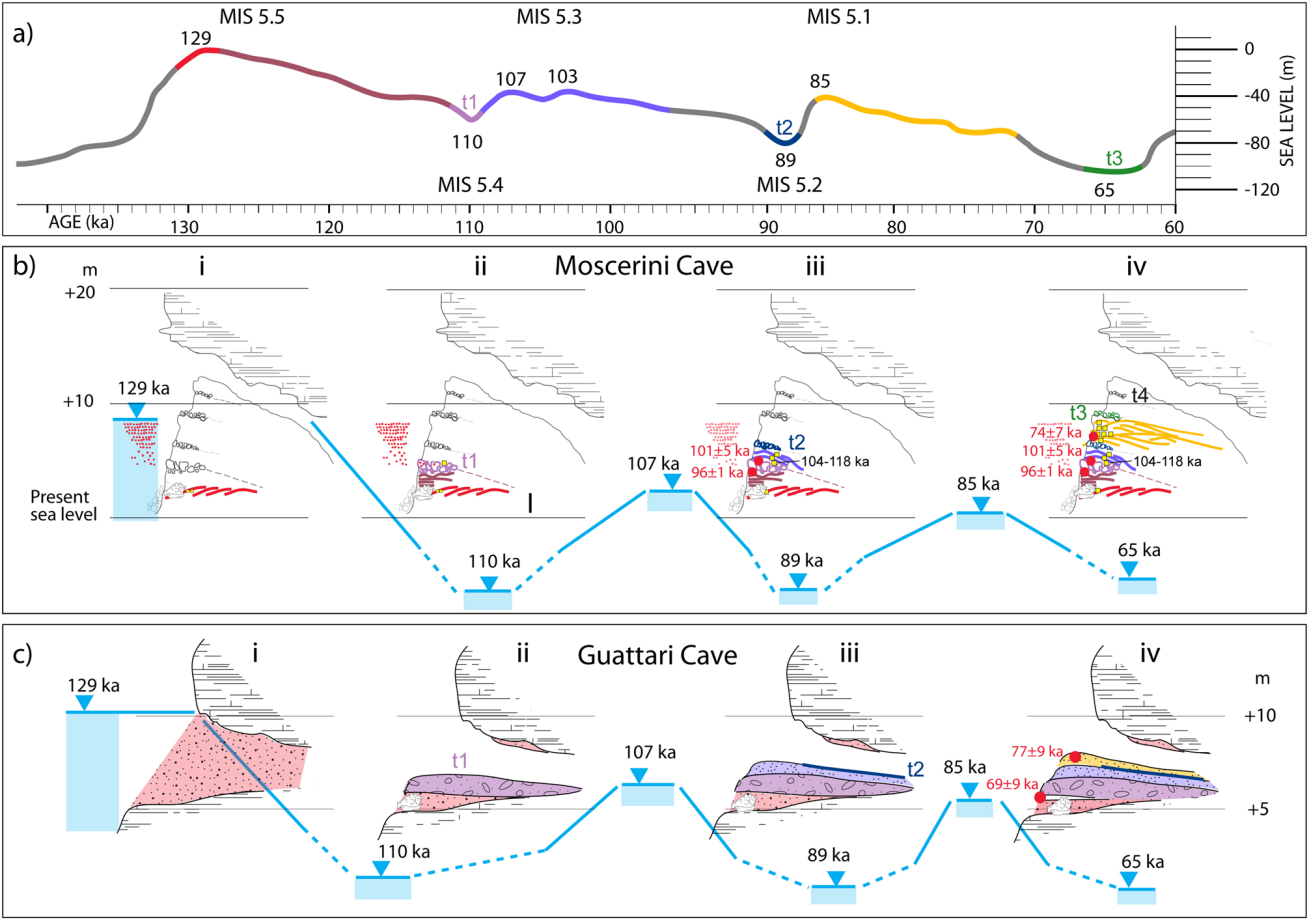
(**a**) Relative Sea Level curve (re-drawn after^43^) reporting the ages for the eustatic events discussed in the reconstruction of the sea-level history at Grotta dei Moscerini (**b**) and Grotta Guattari (**c**). See text for explanations. For colors in this figure please refer to the online version of the paper.

We assume that a maximum sea level at 8 m a.s.l. was reached during MIS 5.5, ca. 129 ka (Fig. 7b–i; red color tract in the relative sea-level curve by^43^ in Fig. 7a). The seawater entered the cave, consistent with the occurrence of the sedimentary package represented by layers #44–#40 (Fig. 7b-iv). These layers include a basal beach deposit (#44) (defined by^31^, as a "Tyrrhenian panchina") rich in pumice clasts, in which *Strombus bubonius* occurs (red color in Fig. 7b-i), followed by brown clays with some charcoal (#43) testifying a dry environment. We correlate the deposition of these deposits with the slow regressive phase (tract in brown of the RSL curve^43^ in Fig. 7a), consistent with the progradational features of the sedimentary wedge of cycle I (Fig. 2a′). The upper deposits (#41, 40) are represented by thin alternating sandy layers and burned or carbon-rich layers, and by stalagmitic crusts (brown color in Fig. 7b-ii). Formation of the thick layer with evidence of human frequentation is correlated instead with the long time span corresponding to the whole regressive phase following highstand of MIS 5.5 (brown tract of the sea-level curve in Fig. 7a), allowing for the accumulation of this horizon. Notably, no pumice is reported to occur in strata #43 through #40.

### Cycle II

The continued sea-level fall reached the lowstand by 110 ka. To this cold period is likely associated emplacement of the crioclastic conglomerate t1 (violet color in Fig. 7b-ii). The ESR-U/Th ages of 96 ± 1–101 ± 5 ka reported by^29^ for the uppermost strata #39 and #38, when considered within the large uncertainties associated with these measures (see Suppl. Mat. 1), as well as with the reference sea-level curve^43^, are substantially in agreement with the proposed chronology. Besides the analytical uncertainties associated with both these ages, it also should be taken into account that the time at which the actual maximum sea level is reached in one region may vary as a function of the local Glacial Isostatic Adjustment (GIA) (e.g.,^44^) and/or other regional factors (e.g.,^45^, and references therein).

Soon after 110 ka, the fast sea-level rise of MIS 5.3 brought the beach to the level of the cave entrance again by 107 ka (Fig. 7b-iii), as the backbeach feature of the pumice-rich deposits constituting layers #37–#30 implies (light blue color in Fig. 7b-iii). However, a maximum sea level lower than the cave floor is suggested by the absence of marine deposits. We remark that possible ages of the pumice collected in layer #37, ranging 118–104 ka, also in light of the fact that the pumice is reworked and these ages should be considered as a *post-quem terminus* for its emplacement, are consistent with the proposed age of 107–103 ka for the set of deposits encompassing layer #37 through #30 (light blue color in Fig. 7a, b-iii).

In order to keep consistency for the proposed sea-level history, we correlate the crioclastic layer t2 with the MIS 5.2 lowstand of 89 ka, at the end of the regressive phase following MIS 5.3 highstand (deep blue color in Fig. 7a, b-iii).

The sea-level approached the cave entrance again by 85 ka, while a new marked sea-level fall began only by 70 ka (yellow tract of the curve in Fig. 7a), consistent with deposition of the most rich in pumice layers #29 through #11, including several intercalations of carbon-rich and stalagmitic horizons (yellow color in Fig. 7b-iv), and with the ESR-U/Th age of 74 ± 7 ka yielded by a tooth collected in layer #26. Finally, a straightforward correlation of the third crioclastic layer t3 with lowstand of MIS 4 (green color in Fig. 7a, b-iv) descends from the proposed chronology of the sea-level oscillations. Moreover, the fourth, uppermost crioclasatic layer t4 can be correlated with the last glacial maximum. Remarkably, no pumice occurs in the strata above t3 despite intense volcanic activity at the Phlegraean Fields and Ischia in the time span 55–12 ka^36^, suggesting that the sea-level never reached close to the cave entrance since then. In other words, we infer that an elevation ≤ 4–6 m is a condition for windblown pumice to get from the beach into the cave, when an uplift of 2–4 m is considered to have occurred at this site in the last 125, by considering the elevation at which the lithophaga burrows stand (8 m) and a sea level 4–8 m higher than in the present for MIS 5.5 highstand.

### Grotta Guattari

A markedly different sea-level history is inferred to have occurred at Grotta Guattari, where the ESR-U/Th age of 69 ± 9 ka obtained from a tooth recovered within the biodetritic layer #7 suggests that this beach deposit is part of the same depositional cycle emplacing the sand layers above, in which another ESR-U/Th age of 79 ± 9 ka was obtained. Moreover,^29^ suggested that the sand deposit rich in shell fragments occurring as incrustation on the roof of the cave was an older beach deposit with respect to that occurring at the base of the sedimentary filling of the cave. Indeed, they interpreted this as an evidence of the fact that the seawaters entered the cave, repeatedly. Such interpretation is consistent with reconstruction in^10^, after which the sea-level was higher than the cave level (located between 5 and 10 m a.s.l., during both MIS 5.5 and MIS 5.3. However, the two biodetritic deposits may be as well part of the same coastal deposit that filled the cave during one highstand, and was then partially eroded after the sea-level drop. This is the interpretation by^21^ and also adopted in^26^, who reported a tidal notch 9.2 m a.s.l. from Grotta Guattari as a sea-level marker of MIS 5.5.

Here we first propose a possible sea-level history for Grotta Guattari which is consistent with that proposed at Grotta dei Moscerini, in the hypothesis that the whole coastal tract between Circeo and Gaeta was substantially stable during the last 125 ka. Then we propose a different interpretation, according the hypothesis by^29^ and with the occurrence of the set of three marine terraces at the Circeo promontory suggested by geomorphological study by^10^ and previously reported in^21^.

We use the elevation of the tidal notch occurring at ca. 9.5 m at Grotta delle Capre^26^ to constrain the maximum sea level during MIS 5.5 at Grotta Guattari (Fig. 7c-i). At this stage the seawaters entered the cave and released the beach deposits which filled the cave, completely.

Their erosion occurred during the following regressive phase culminating in the glacial lowstand of MIS 5.4, during which the thick conglomeratic layer (analog of the crioclastic layer t1 at Grotta dei Moscerni) emplaced (Fig. 7c-ii). A first dune deposit^18^ emplaced during the successive highstand of MIS 5.3, when the sea level approached the cave entrance again (Fig. 7c-iii). The carbonatic crust on top of this first dune deposit (SU 4 in Fig. 2b) may be regarded as the corresponding evidence for the occurrence of a dry and cold period during MIS 5.2, as the t2 layer in Grotta dei Moscerini (Fig. 7c-iii).

Finally, another dune deposit emplaced during MIS 5.1 highstand, consistent with ESR-U/Th age of 77 ± 9 ka yielded by the tooth enamel of the specimen collected in layer #5 (SU 3 in Fig. 2b). We remark that the exact position of the *Bos* tooth yielding 69 ± 9 ka is uncertain since it was collected for analysis by^29^ at the IsIPU repository in Anagni, who referred it to layer #7 according to the stratigraphy by^2^. However, its clearly misplaced occurrence within a beach deposit strongly suggests, in our opinion, that it may have occurred in the upper, loose sandy layer on top of it, that we have highlighted in our re-excavated section (SU 9 in Fig. 4c). This layer may be regarded as a later filling of a void originally occurring between the beach deposit and the base of the conglomeratic layer above. This fact suggests that the tooth was introduced in the small cavity later on, during the regressive phase following MIS 5.1, consistent with its age of ca. 69 ka (Fig. 7c-iv).

**Figure 8:**
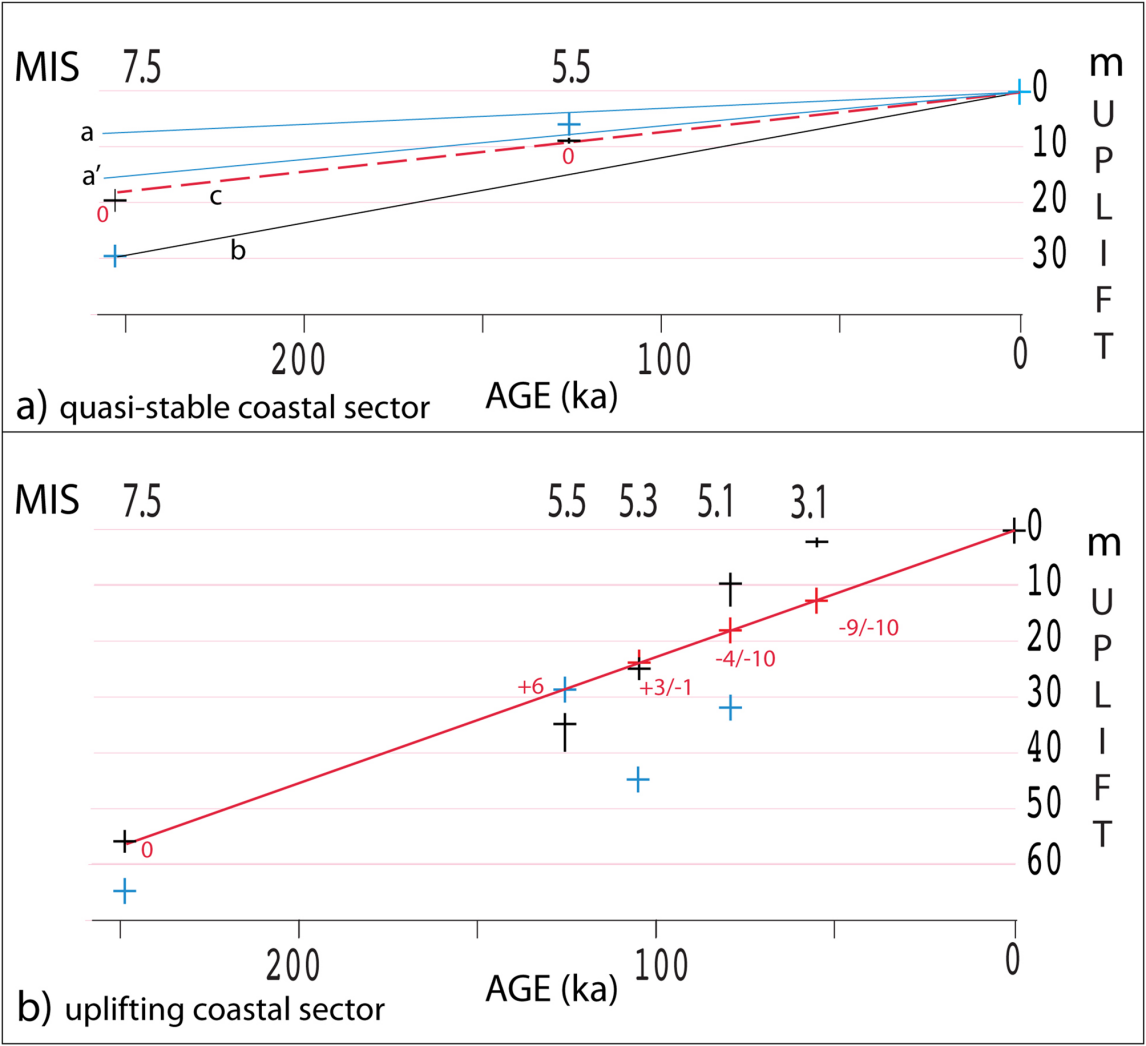
(**a**) Uplift curves based on sea-level elevations (black crosses) correlated with MIS 5.5 and MIS 7.5 inferred by tidal notches at 9.6 and 20 m a.s.l. occurring at the Circeo promontory^21,26^ ; blue crosses: sea-level correction according to current literature^12^. (**b**) Uplift curve based on sea-level elevations (black crosses; vertical bar includes the associated error) correlated with MIS 3.1 through MIS 7.5 inferred from the marine terraces of the Latium coast^10^; red crosses: sea-level elevation after correction for the absolute maximum sea level at each interglacial calculated assuming a constant uplift rate during the last 250 ka; blue crosses: sea-level correction according to current literature^12^. See text for explanation.

While the sea-level history reconstructed above seems consistent with the observed stratigraphy and available geochronologic constraints, it does not provide explanation for the tidal notch occurring 20 m a.s.l. on the Circeo reef and the associated terrace (SL1 and T1 in Fig. 1c, d, respectively), as reported in^21^. Indeed, also admitting that a weak uplift which justifies the occurrence of the MIS 5.5 coastline at ca. + 9 m instead that 4–6 m above the present day acted in this region, it would not be enough to explain a MIS 7 sea level at + 20 m. The graphic in Fig. 8 shows the range of uplift rate (a, a′) associated with a MIS 5.5 paleo-sea level at 9.6 ± 0.5 m (black cross), when a correction for a maximum sea level 4–8 m higher 125 ka than in the present (blue cross) is considered. In contrast, a much higher uplift rate (b in Fig. 8a) is required in order to have the paleo-coastline of MIS 7.5 at + 20 m (black cross), when a correction of − 10 m (blue cross) for the maximum sea level of 250 ka^12^ is considered. However, we remark that assuming that at both MIS 7.5 and MIS 5.5 the maximum sea level was equal to the Present (i.e., providing no sea-level correction in Fig. 8a) a constant moderate uplift is obtained during the last 250 ka (red dashed line c in Fig. 8a). However, such assumptions, besides seeming unlikely for MIS 5.5 for which univocal indications of a sea level significantly higher than in the Present exists (e.g.,^5^), could not match the paleo-sea level elevations observed in the uplifting sector assuming a constant uplift rate (Fig. 8b).

Therefore, in contrast with this first, conservative hypothesis, we also consider an alternative interpretation that accounts for the occurrence of an upper, subdued marine terrace at 40–50 m, combined with the terraces associated with the tidal notch of 20 m and 9 m, matching the elevation of the three terraces reconstructed between Anzio and Circeo by^10^.

Indeed, this coincidence of terrace elevations suggests that the possibility that this coastal reach, unlike the southernmost one where Grotta dei Moscerini is located, experienced ca. 30 m uplift in the last 125 ka cannot be excluded.

**Figure 9:**
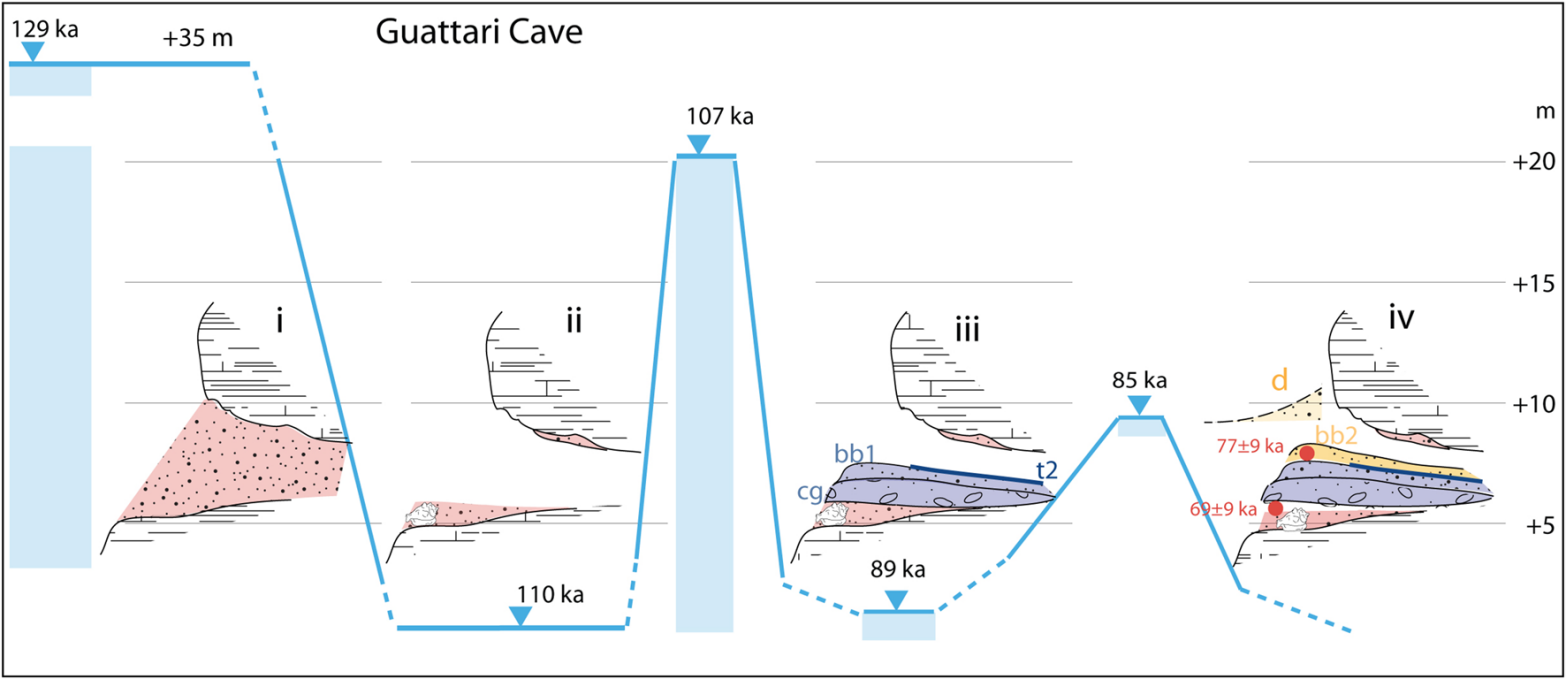
Alternative sea-level history for Grotta Guattari. See text for explanations.

An identical reconstruction as that provided in Fig. 7b in the time span 129–107 ka is given for the sea-level history at Grotta Guattari in Fig. 9, except assuming that the maximum sea level during MIS 5.5 and MIS 5.3 reached up to 35 m and 20 m a.s.l., respectively (Fig. 9i-ii). Afterwards, we hypothesize that the thick conglomeratic layer made of cemented, rounded blocks (cg in Fig. 9-iii) may have formed in consequence of the wave action causing the disruption of the cave vault during the periods in which the seawater entered the cave, while the backbeach deposit bb1 and the carbonatic crust t2 have the same origin as in the previous reconstruction, being originated during the regressive phase leading to MIS 5.4 lowstand. Finally, the upper dune deposit bb2 was emplaced during MIS 5.1 highstand, since 85 ka through 70 ka. The lack of more marine or beach deposits within the cave seems conflicting with the occurrence of a tidal notch at ca. 9 m at the nearby Grotta delle Capre which, in the case of this alternative hypothesis, must correlate MIS 5.1. However, inundation of the cave might easily have been hindered by the presence of a backbeach dune ridge (d in Fig. 9-iv), successively partially removed by erosion. Indeed, no tidal notch has been observed on the front face of Guattari cave during the new survey. Erosion during the regressive phase following MIS 5.3 may be also regarded as the cause of the opening of a smaller cavity at the base of the conglomeratic layer cg, at around 69 ± 9 ka (Fig. 9-iv).

### Glacio-eustatic and paleo-climatic implications

Assuming that a homogeneous uplift rate of 0.24 mm/year characterized the coast of Latium during the last 125 ka, Marra et al.^10^ have calculated corresponding sea levels of + 6 m, + 3/ − 1 m, and − 4/ − 10 m a.s.l. for MIS 5.5, MIS 5.3 and MIS 5.1, respectively, based on corresponding marine terrace elevations (Fig. 8b). Data for the two youngest highstands differ significantly from coral and speleothem- based sea-level markers, as well as with δ^18^O derived estimations (see^12^ for a review), indicating values of ca. + 4/ + 8 m for MIS 5.5 sea level, and of ca. − 20 m/ − 25 m for MIS 5.3 and MIS 5.1 sea level with respect to the present.

In the present work, through the detailed re-analysis of the sedimentary record, the sea-level markers, ESR-U/Th ages, and pumice geochemistry from Grotta dei Moscerini we outline that the sea-level markers indicating a paleo-coastline at ca. + 8 m correlate well with the MIS 5.5 age proposed in previous literature^5–7,26^, in the hypothesis that this coastal sector did not undergo to a sensible tectonic uplift during the last 125 ka. However, as already remarked in^10^, the same elements clearly indicate that the sea-level during MIS 5.3 and MIS 5.1 was very close to the cave entrance located ~ 3 m a.s.l., consistent with the corresponding sea levels calculated in the northern, uplifted coastal sector (Fig. 8b).

Moreover, as reported by^10,21^, *Strombus bubonius* occurs on two different terraces (i.e. at 24–27 m and 11–17 between Argentario and the Tiber mouth, and at 5–12 m and 2–3 m between Anzio and Circeo), suggesting either widespread reworking or that the Senegalese fauna persisted in this region which possibly acted as *refugium* for such exotic species. These data from the Latium coast are consistent with observations at the Balearic Islands accounting for occurrence of *Strombus bubonius* on different terraces and for evidence of a sea level at similar elevation during MIS 5.5 and either MIS 5.3 or MIS 5.1^13,14,46–49^, suggesting that these anomalous elevations for the sea level during the two substages of MIS 5.3 and 5.1 are a regional feature.

The lowest terrace at 2–3 m remains an open question, at the moment. Its presence in the northernmost coastal sector^24^ as well as south of Anzio through the Circeo^19,21^ is a further indication suggesting that this whole coastal sector was homogeneously subjected to the same uplift rate 250 ka through the Present. Regarding its age, a simple geometric principle suggests that this lowest terrace correlates the sea-level highstand of MIS 3, occurred ca. 55 ka (Fig. 8b). However, such correlation would imply a sensibly higher sea level for this highstand with respect to the global estimations based on the δ^18^O benthic record (i.e., ca. − 10 m instead of ca. − 60 m^12^).

### Implications on the Neanderthal frequentation of the caves

A remarkable coincidence that deserves to be discussed, is that the age determinations on vertebrate teeth for Grotta dei Moscerini (96 ± 1–101 ± 5 ka, and 74 ± 7 ka) match the timing of the two highstands of MIS 5.3 and 5.1 occurred around 100 ka and 80 ka. This fact suggests that human frequentation of this cave (to which the presence of vertebrate teeth should also be associated) was concentrated in the periods during which the cave entrance faced a shore and, nevertheless, very likely it was not accessible walking along the coast because of the discontinuous presence of small beach reaches at the foot of the steep carbonatic cliff. A condition offering ideal repair as well as access to a resource for food and an uncommon material to produce tools: bivalves and their shell. Indeed, according to^4^, the "Neanderthals on the beach" of Moscerini cave used to fish shells to provide them with food, besides using the valves to produce artifacts. However, unlike the occurrence also outlined by these authors in Grotta Santa Lucia, located in northern Italy ca. 200 m a.s.l. and ca. 3.5 km far from the coast, we remark that the abundant, small pumice clasts occurring in Grotta dei Moscerini are part of the sedimentary filling of the cave. In our opinion, there is no objective mean to prove that they were collected on the beach by the Neanderthals. In contrast, it may be possible that some of the largest ones, like that labeled #37 (Fig. 3), which occurred immediately above the thick successions of layers with traces of frequentation (#39, #38), or also the almost spherical T4 (Fig. 3) found in the interior excavation, may have been collected and brought inside the cave as a consequence of the human curiosity.

In contrast, the scanty lithic assemblage recovered at Grotta Guattari, essentially concentrated in the upper part of layer SU 5 (Fig. 2b) correlated with MIS 5.3 highstand, along with the abundant faunal remains occurring in layer SU 3 correlated with MIS 5.1 (unpublished data acquired during the ongoing "Guattari Project"), as well as the occurrence of the Neanderthal skull above the ground floor, are possible indication that the cave was repeatedly submerged during MIS 5.5 and MIS 5.3 highstands, and became easily accessible only since the regressive phase following MIS 5.3, initiated 100 ka.

## Conclusions

Results of this study provide new evidence supporting the alternative hypothesis made by^10^ on the tectonic stability of the coastal reach south of Circeo promontory, implying a MIS 5.5 age for the sea-level markers occurring ~ 8 m a.s.l. at Grotta dei Moscerini, and for sea levels only few meters below this elevation during MIS 5.3 and MIS 5.1 highstands.

This new scenario implies very different paleogeographic and environmental conditions is this area as well as in the whole Mediterranean coastal regions than previously thought, and has also relevant consequences on the time of frequentation and on the modality of access by Neanderthals to the caves of southern Latium, as discussed in the final section of the present paper. Finally, this work provides new elements to geographically constrain the extension of the area subjected to uplift, with important geodynamic implications on the volcanotectonic and subduction processes in this region.

The causes of the large mismatch between the sea levels during MIS 5.3 and 5.1 in the Mediterranean Sea with respect to alleged global values have been discussed in^10^ who remarked that the datum from the Latium coast may be regarded as suggesting that previously unforeseen GIA effects affected the Mediterranean Region during MIS 5.3 and 5.1. At the same time, the necessity to invoke similar isostatic responses in very different geodynamic and geographic regions in order to explain the same sea level estimates for MIS 5.5, 5.3 and 5.1, is challenging, and the observed sea-levels may have different causes, which might be investigated through the re-evaluation of the ice-models proposed so far and the estimation of global ice volume based on the δ^18^O benthic record.

## Supplementary information

Supplementary information

## Data Availability

All data generated or analyzed during this study are included in this published article.
