# Supplementary material for: Anomalous Last Interglacial Tyrrhenian sea levels and Neanderthal settling at Guattari and Moscerini caves (central Italy)
Source: Sci Rep. 2020 Jul 17;10:11929. doi: 10.1038/s41598-020-68604-z (PMC7368079; doi:10.1038/s41598-020-68604-z)
Supplement: Supplementary file 1 — Supplementary information [file 41598_2020_68604_MOESM1_ESM.doc]

**Anomalous Last Interglacial Tyrrhenian sea levels and Neanderthal settling at Guattari and Moscerini caves (central Italy)**

Marra F.^1^, Rolfo M.F.^2^, Gaeta M.^3^, Florindo F.^1,4^

1) Istituto Nazionale di Geofisica e Vulcanologia, Via di Vigna Murata 605, 00143 Rome, Italy

2) University of Rome “Tor Vergata”, - Department of History, Humanities and Society, Via Columbia 1, 00133 Roma  - Italy

3) Dipartimento di Scienze della Terra, “Sapienza” Università di Roma, Piazzale Aldo Moro 5, 00185 Roma, Italy

4) Institute for Climate Change Solutions, via Sorchio, 61040-Frontone, Pesaro e Urbino

**Supplementary Material**

**A) Geochronologic and biochronologic markers**

**Senegalese fauna**

Occurrence of the "warm guest" *Strombus bubonius* LAMARCK, 1892 (synonym of *Peristrombus latus* GMELIN, 1791), a gastropod part of the "Senegalese fauna" which entered the Mediterranean Sea from Gibraltar and spread over its southern and western coasts [1], has been considered exclusive of the Last Interglacial sea-level highstand (i.e., MIS 5.5, ca. 125 ka), which the Tyrrhenian marine stage was associated to [2]. This conviction was based on the unusually warm temperature characterizing MIS 5.5, resulting into a maximum sea-level several meters higher than in the Present, creating the unique paleoclimatic and paleogeographic conditions allowing for the diffusion of this exotic marine fauna (see [3] for a review). However, already [4] suggested the occurrence of several *Strombus* levels in the Mediterranean Sea, a concept which was re-proposed and supported by [5] who, based on the studies by [6, 7, 8], reported the occurrence of three *Strombus* levels along the Latium coast of the Tyrrhenian Sea. According to the Authors, these deposits form three terraces at progressively higher elevation of 2-3 m, 10-15 m and 18-25 m along the northern Latium coast, which were referred as 1st, 2nd and 3rd *Strombus* level [5, 9].

**Aminoacid ratios**

In contrast with previous view, [10] rejected the interpretation of three *Strombus* levels, stating that multiple occurrences should be attributed to re-deposition of misplaced specimen coming from reworking of in situ fauna occurring in the upper terrace. These authors supported their assumption by defining a distinctive geochemical fingerprint for MIS 5.5, based on particular amino acid ratios determined on mollusk shells (i.e., isoleucine epimerization). Four aminozones were empirically established, characterized by mean ratios ranging 0.30 (aminozone C/D), 0.40 (E), 0.50 (F) and 0.60 (G). Aminozone E, despite the lack of available unambiguous radiometric ages, was considered the marker of MIS 5.5 interglacial, while aminozone G was suggested to correlate to MIS 9 (ca. 320 ka). By consequence, aminozone F should correspond to MIS 7 (250-200 ka) and C/D to MIS 5.3/5.1 (ca. 100 and ca. 80 ka, respectively).

However, it must be noted that controversial results deriving from application of this dating method were discussed in the literature (e.g. [11, 12, 13]), questioning its reliability. Moreover, occurrence of *S. bubonius* has been reported on the MIS 7 terraces at several locations in the Mediterranean Sea (e.g., [14] and references therein), excluding that it may represent displaced specimens from an older, MIS 5.5 terrace. In these cases, incorrect age attribution to the terraces has been claimed by the supporters of a unique MIS 5.5 presence for *S. bubonius* and the associated Senegalese fauna (e.g., [15]).

**ESR/U-Th ages**

A widely adopted and tested dating method that has been applied to the context of marine terraces in Latium is the Electron Spin Resonance (ESR) coupled with the U-Th method, usually used to date herbivorous teeth [16]. The combination of U-series and ESR analyses can be used also to date mollusk shells, which are well known to be open-systems experiencing post-depositional U-uptakes (e.g., 17, 18), through the determination of an U-uptake parameter p allowing to calculate a corrected ESR/U-series age. Reliability of this latter application of the method has been questioned (e.g., [15]), however results from Quadrato site (Fig. S1e) for the deposit of the 11-17 m coastal terrace in [19] are closely consistent with those on tooth enamel for the equivalent deposits in Grotta dei Moscerini and Grotta Guattari [20, 21] (Table 1) (Fig S1e), supporting reliability of all these ages.

| a) Grotta Guattari | | | | |
| --- | --- | --- | --- | --- |
| Schwarz et al., 1990/91 | | | | This work |
| LAYER | SAMPLE | LU age (ka) | average age | weighted mean age |
| 5 | GG TrBeHa | 87.4±7.1 | 77.5±9.5 | 77.6±9.4 |
|  | GG TrBeHb | 82.9±9.4 |  |  |
|  | GG TrBeHc | 84.7±17.2 |  |  |
|  | GG TrBeHd | 77.2±4.8 |  |  |
|  | GG TrBeIa | 63.5±8.1 |  |  |
|  | GG TrBeIb | 69.0±15.5 |  |  |
| 7  (beach) | GG TrBa | 69.6±8.0 | 69.0±2.4* | 69.2±9.0 |
|  | GG TrBb | 67.7±10.3 |  |  |
|  | GG TrBc | 72.1±7.9 |  |  |
|  | GG TrBd | 66.6±10.1 |  |  |

| b) Grotta dei Moscerini | | | | |
| --- | --- | --- | --- | --- |
| Schwarz et al., 1989 | | | | This work |
| LAYER | SAMPLE | LU age (ka) | average age | geometric mean age |
| 25 | MO 25 | 79 | - | 75.9 |
| 26 | MO 26a | 67 | 74±7 |  |
|  | MO 26b | 81 | - |  |
| 33 | MO 33a | 69 | 106±17 | - |
|  | MO 33b | 123 |  |  |
| 35 | MO 35 | 66 | - | - |
| 38 | MO 38a | 96 | 101±5 | 98.7 |
|  | MO38b | 106 |  |  |
| 39 | MO 39a | 97 | 96±1 |  |
|  | MO39b | 96 |  |  |

TABLE 1 - ESR/U-Th ages on tooth enamel performed at Grotta Guattari and Grotta dei Moscerini. *The small error associated with this measure (±2.4) is inconsistent with data and is evidently a typos in the table by Schwarcz et al., 1990/91.

At Quadrato, a Glycimeris shell recovered at ca. 10 m a.s.l. within a beach ridge sand deposit yielded an ESR/U-series age of 79±7 ka [19].

At Grotta Guattari, two teeth from layer #5 (dune sand above the beach layer, see Fig. S1e) gave an average age of 77±9 ka. A single tooth sample from layer #7 gave an average age of 69±9 ka (Table 1a).

At Grotta dei Moscerini, ESR/U-series ages were calculated without an associated error, due to the lack of precise environmental dose rate (the dated teeth were collected from the materials stored at the IsIPU repository and no in situ dosimetric measure was performed), therefore ages should be regarded with caution. These ages were reported indeed with an error interval which represents the full spread with respect to the simple arithmetic mean among the different measures reported in the third column of Table 1b. We have re-calculated two geometric mean ages of 75.9 ka and 98.7 ka for layers 25/26 and 38/39, respectively. However, in Figure S1d and in the main text we have assumed the age of 74±7 for layer 26 and those of 101±5 and 96±1 (which provide a weighted mean age of 96±1 ka and are therefore statistically indistinguishable) for layers 38-39 reported by Schwarz et al. (1989), while we do not take into account the poorly constrained age for layer 33 and the stratigraphically inconsistent age for layer 35.

***Dama dama dama***

Biochronologic constraints provided by the occurrence of *Dama dama dama* within the fossil assemblage recovered at Campoverde from a sedimentary succession forming the 31-40 m terrace extending from Anzio to Circeo promontories (Fig. S1b) evidenced a maximum oldest age within MIS 5.5 [22]. Following this assumption, the two lowest terraces at 20-28 and 11-17 m have to be younger and should correlate with later interglacials (i.e., MIS 5.3 and MIS 5.1). Indeed, as remarked in [19], one of the most relevant features from the biochronological point of view of the *Cervidae* family and the *Dama dama* species in particular, is the great and fast adaptability to different environments*,* which derives from a high phenotypic plasticity; this causes a rapid development over time of the morphological characteristics that are reflected on the rapid appearance of morphologically and genetically distinct populations recognizable at the level of subspecies [23]. In order to allow the evolutionary differentiations that will cause the formation of different populations and, then, the appearance of different subspecies, the individuals must live in completely separate areas, resulting in the fact that different subspecies cannot inhabit the same territory. Therefore, the red deer subspecies *D. d. tiberina* and *D. d. dama* represent good chronologic markers of the Middle-Late Pleistocene boundary for central Italy since they are present in limited intervals, very well constrained by ^40^Ar/^39^Ar ages, corresponding to MIS 8.5 through 7, and to MIS 5.5 through MIS 2, respectively [24].


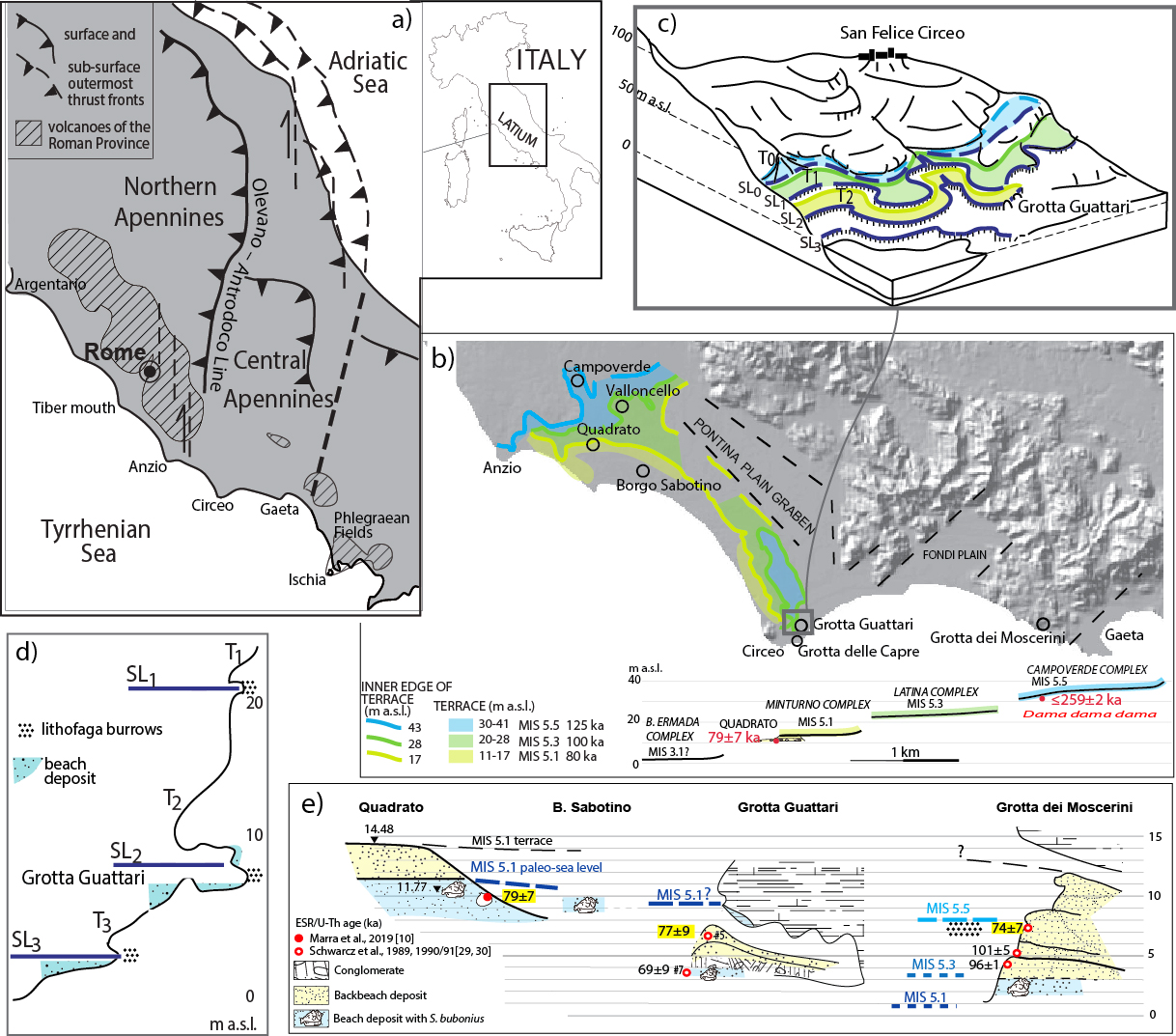
Figure S1 -a) Structural sketch of central Italy. b) Digital Elevation Map (DEM) image of the southern Latium coast showing the suite of three marine terraces described in [19]. Background DEM: TINITALY/01 square WA 6570, used with permission by the Istituto Nazionale di Geofisica e Vulcanologia, Rome. The cross-section shows the correlation among the marine terraces reconstructed by [25] and those identified by [10]; bio- and geochronologic constraints (in red) allowing for correlation with MIS 5.5, MIS 5.3 and MIS 5.1 are also reported. c) coastlines (SL) and related terraces (T) reconstructed at Circeo promontory in the surrounding of Grotta Guattari by [26, 27]. d) sea-level indicators represented by tidal notches, lithophaga burrows and biodetritic beach deposits. e) Correlation among different sea-level markers ranging 10-8 m a.s.l., represented by the terraced deposit of the Minturno complex in Quadrato, and by the beach to backbeach sedimentary filling of Grotta Guattari and Grotta dei Moscerini; the three consistent ESR/U-Th ages suggesting correlation with MIS 5.1 are shown. All drawings by F. Marra.

**B) Geo- and biochronologic constraints to the marine terraces in southern Latium coast**

**31-40 m terrace (Campoverde complex)**

^40^Ar/^39^Ar dating of a volcanic deposit adhering to a bone fragment recovered in Campoverde (Fig. S1b) provided post-quem age of 259±2 ka [19], implying a minimum MIS 7 age for the 31-40 m terrace. Moreover, the occurrence of several remains of *Dama dama dama* in the fossil assemblage of Campoverde, combined with sedimentological-stratigraphical analysis of the succession forming the 31-40 m terrace, allowed [22] to exclude an age older than MIS 5.5 (125 ka) for it. The age of 259±2 ka confirmed the occurrence of reworked specimens in the fossil record of Campoverde outlined by paleontological analysis in [22] and constrained their age within MIS 8.5/7.5 (285 - 245 ka). Indeed, these authors evidenced the presence of two faunal assemblages: an older one with *Mammuthus ex gr. chosaricus-primigenius and D. dama cf. D. dama tiberina*, referred to MIS 8.5/7, and a younger one, characterized by the sub-species *D. d. dama*, whose presence in peninsular Italy is limited to the interval MIS 5.5 - MIS 2 [19, 23].

**20-28 terrace (Latina complex)**

The deposits forming the intermediate terrace have been described by Marra et al. [19, 22] at Valloncello, a site located on the inner margin of the 20-28 paleo-surface (Fig. S1b). They are characterized by the occurrence at 25.4 m a.s.l. of a whitish concretionary carbonatic deposit, 50 cm in thickness, overlying barren grey-yellowish clay. This clay deposit correlates well with the layer of greenish clay to sandy clay loam, 1.5-3.0 m in thickness, described by [25] to occur in the more flat and less eroded, upper part of the Latina complex, at ca. 25 m a.s.l.. Along with the overlying calcareous mud, the Valloncello succession matches the lagoon to coastal alluvial plain facies of the Latina complex, providing a paleo sea-level marker at ca. 25 m a.s.l. (SL1 in Figure 1c-d).

The faunal assemblage from Valloncello re-analyzed in [19] did not provide any useful chronologic constraints. Similarly, a sample of the volcanic material adhering to a lithic instrument previously recovered in this area yielded age of 348±4 ka [19], providing only a post-quem terminus for the overlying sedimentary deposits.

**11-7 m terrace (Minturno complex)**

Stratigraphic investigation performed at Quadrato (Fig. S1b) allowed [19, 22] at identifying a regressive surface at ca. 12 m a.s.l. associated with this terrace (Fig. S1e). A few rather abraded *Strombus* specimens, as opposed to a large number of very well preserved *Glycimeris*, along with *Cerastoderma* and rare *Pecten* and *Conus* were recovered during the field survey.

The ESR/U-series age of 79±4 ka yielded by one *Glycimeris* confirmed previous assumption of a MIS 5.1 age for the 11-17 m paleo-surface and the associated ~12 m sea level (SL2 in Fig. S1c-d). In contrast, the U-series age of 198±8 ka performed on a coral specimen recovered by [28] at Quadrato suggested that it is from a reworked, older unit. Moreover, two groups of AIle/Ile ratios yielded by mollusk shells from this site were previously reported in [10], accounting for widespread reworking affecting the deposits of this terrace.

REFERENCES

1. Gignux, M., 1913. Les formations marines pliocènes et quaternaires de l’Italie du Sud et de la Sicilie. Ann. Univ. Lyon, 36, 693 pp.
2. Issel, A., 1914. Lembi fossiliferi quaternari e recenti osservati nella Sardegna meridionale dal Prof. D. Lovisato, Rend. R. Acc. Lincei 23.
3. Antonioli, F., Lo Presti, V., Rovere, A., Ferranti, L., Anzidei, M., Furlani, S., Mastronuzzi, G., Orru, P.E., Scicchitano, G., Sannino, G., Spampinato, C.R., Pagliarulo, R., Deiana, G., de Sabata, E., Sanso, P., Vacchi, M., Vecchio, A., 2015. Tidal notches in Mediterranean Sea: a comprehensive analysis. Quat. Sci. Rev. 119, 66-84. https://doi.org/10.1016/j.quascirev.2015.03.016
4. Bonifay, F., Mars, P., 1959. Le Tyrrhénien dans la cadre de la chronologie quaternaire méditerranéenne, Bull. Soc. Geol. Fr. 1(7).
5. Ambrosetti, P., Azzaroli, A., Bonadonna, F.P., Follieri, M., 1972. [A scheme of Pleistocene chronology for the Tyrrhenian side of central Italy](http://serials.unibo.it/cgi-ser/start/it/spogli/df-s.tcl?prog_art=8930723&language=ITALIANO&view=articoli), Boll. Soc. Geol. It. 91(1), 169-184.
6. Bonadonna, F.P., 1967a. Studi sul Pleistocene del Lazio. III. Linee di costa lungo il litorale di Tarquinia (Lazio settentrionale), Geol. Rom. 6, 121-135.
7. Bonadonna, F.P., 1967b. Studi sul Pleistocene del Lazio IV. La linea di costa tirreniana di Ponte Galeria (Roma), Quaternaria 9, 285-299.
8. [Bonadonna, F.P., Bigazzi, G., 1970. Studi sul Pleistocene del Lazio. VIII. Datazione di tufi intertirreniani della zona di Cerveteri (Roma) mediante il metodo delle tracce di fissione, Boll. Soc. Geol. It. 89(04), 463-473](http://serials.unibo.it/cgi-ser/start/it/spogli/df-s.tcl?prog_art=8930820&language=ITALIANO&view=articoli).
9. Bigazzi, G., Bonadonna, F.P., Iaccarino, S., 1973. Geochronological hypothesis on Plio-Pleistocene boundary in Latium Region (Italy). Boll. Soc. Geol. It. 92, 341-422.
10. Hearty, P.J., Dai-Pra, G., 1986. Aminostratigraphy of Quaternary marine deposits in the Lazio region of central Italy, in: Ozer, A., Vita-Finzi, C. (Eds.), Dating Mediterranean shorelines, Zeitschrift fuer Geomorphologie, Supplementband 62, 131-140.
11. Radtke, U. 1983. Genese und Artestellung der marinen terrassen swischen Civitavecchia und Monte Argentario (Mittelitalien) unter besonderer Berucksichtigung der Elektronespin-Resonanz-Alters-bestimmungsmethod. Dusseldorferer Geographische Schriften 22, 178-184.
12. Radtke, U., 1986. Value and risks of radiometric dating of shorelines. Geomorphological and Geochronological investigations in Central Italy, Eolian Islands and Ustica (Sicily). Zeitschrift für Geomorphologie 62, 167-181.
13. Bartolini C. Bosi, C., Belluomini, G., 1984. Isoleucine epimerization as a tool for dating Northern Latium raised beaches. Boll. Soc. Geol. It. 103, 485-490.
14. Zazo, C., Goy, J.L., Dabrio, C.J., Lario, J., González-Delgado, J.A., Bardají, T., Hillaire-Marcel, C., Cabero, A., Ghaleb, B., Borja, F., Silva, P.G., Roquero, E., Soler, V., 2013. Retracing the Quaternary history of sea-level changes in the Spanish Mediterranean-Atlantic coasts: geomorphological and sedimentological approach. Geomorphology 196, 36–49.
15. Muhs, D.R., Simmons, K.R., Meco, J., Porat, N., 2015. Uranium-series ages of fossil corals from Mallorca, Spain: The “Neotyrrhenian” high stand of the Mediterranean Sea revisited. Palaeogeography, Palaeoclimatology, Palaeoecology 438, 408–424.
16. Grün, R., Schwarcz, H.P., Chadam, J.M., 1988. ESR dating of tooth enamel: coupled correction for U-uptake and U-series disequilibrium. Nuclear Tracks and Radiation Measurements, 14, 237-241.
17. Kaufman, A., Broecker, W.S., Ku, T.L., Thurber, D.L., 1971. The status of U-series methods of mollusk dating. Geochimica et Cosmochimica Acta, 35, 1155-l 183.
18. McLaren S.J., Rowe P.J., 1996. The reliability of uranium-series mollusc dates from the Western Mediterranean Basin. Quaternary Science Reviews, 15, 709-717.
19. Marra, F., Bahain, J.J., Jicha, B., Nomade, S., Palladino, D.M., Pereira, A., Tolomei, C., Voinchet, P., Anzidei, M., Aureli, D., Ceruleo, P., Falguères, C., Florindo, F., Gatta, M., Ghaleb, B., La Rosa, M., Peretto, C., Petronio, C., Rocca, R., Rolfo, M.F., Salari, L., Smedile, A., Tombret, O., 2019. Reconstruction of the MIS 5.5, 5.3 and 5.1 coastal terraces in Latium (central Italy): a re-evaluation of the sea-level history in the Mediterranean Sea during the Last Interglacial, Quaternary International. DOI:10.1016/j.quaint.2019.09.001
20. Schwarcz, H. P., et al. Reexamination of Grotta Guattari: Uranium-Series and Electron-Spin-Resonance Dates. *Current Anthropology* **32**, 3, 313-316 (1989).
21. Schwarz, H.P., Buhay, W., Grün, R., Stiner, M., Kuhn, S., Miller, G.H., 1990-91. Absolute dating of sites in coastal Lazio. Quaternaria Nova 1, 51-67.
22. Marra, F., Petronio, C., Ceruleo, P., Di Stefano, G., Florindo, F., Gatta, M., La Rosa, M., Rolfo, M.F., Salari, L., 2018. The archaeological ensemble from Campoverde (Agro Pontino, central Italy): new constraints on the Last Interglacial sea level markers. Scientific Reports, SREP-18-23649, DOI : 10.1038/s41598-018-36111-x
23. Di Stefano, G., Petronio, C., 1997. Origin and evolution of the European fallow deer (*Dama*, Pleistocene). Neues Jahrbuch für Geologie und Paläontologie Abhandlungen 203, 57-75.
24. Marra F., Nomade S., Pereira A., Petronio C., Salari L., Sottili G., Bahain J.J., Boschian G., Di Stefano G., Falgueres C., Florindo F., Gaeta M. Giaccio B., Masotta M., 2018b. A review of the geologic sections and the faunal assemblages of Aurelian Mammal Age of Latium (Italy) in the light of a new chronostratigraphic framework. Quaternary Science Reviews 181, 173-199. doi.org/10.1016/j.quascirev.2017.12.007
25. Sevink, J., Vos, P., Westerhoff, W.E., Stierman, A., Kamermans, H., 1982. A sequence of marine terraces near Latina (Agro Pontino, central Italy), Catena 9, 361-368.
26. Durante, S., Settepassi, F., 1974. Livelli marini e molluschi tirreniani alla Grotta delle Capre (Circeo). Mem. Ist. Pal. Um. 2, 286-296.
27. Durante, S., Settepassi, F., 1976-77. Malacofauna e livelli marini tirreniani a Grotta Guattari, Monte Circeo (Latina). Quaternaria 19, 35-69.
28. Liboni, A., 1983. Affioramento fossile con malacofauna tra il Quadrato e Casale Nuovo (Borgo Sabotino - Latina), Studi per l'ecologia del Quaternario, 131-134.
